# Pathogenic Foodborne Bacteria in the Game Meat of Hunted Large Ungulates—A Systematic Review and Meta‐Analysis

**DOI:** 10.1111/1541-4337.70657

**Published:** 2026-09-18

**Authors:** Maria Fredriksson‐Ahomaa, Silvia Bonardi, Bozena Futoma‐Koloch, Ioannis Sakaridis, Jelena Petrovic, Inge Van Damme

**Affiliations:** ^1^ Department of Food Hygiene and Environmental Health, Faculty of Veterinary Medicine University of Helsinki Helsinki Finland; ^2^ Inspection of Food of Animal Origin Unit, Department of Veterinary Science University of Parma Parma Italy; ^3^ Department of Microbiology, Faculty of Biological Sciences University of Wroclaw Wroclaw Poland; ^4^ Veterinary Research Institute, Hellenic Agricultural Organization ‐ Dimitra, Campus of Thermi Thessaloniki Greece; ^5^ Department for Food and Drugs Examination Scientific Veterinary Institute Novi Sad Novi Sad Serbia; ^6^ Department of Translational Physiology, Infectiology and Public Health Ghent University Merelbeke Belgium; ^7^ Service of Foodborne Pathogens, Sciensano Brussels Belgium

**Keywords:** *Campylobacter*, foodborne pathogenic bacteria, game meat safety, hunted ungulates, *Listeria*, *Salmonella*, STEC, Yersinia

## Abstract

Zoonotic bacterial pathogens are a major concern for food safety, yet information regarding their occurrence in game meat remains fragmented. This systematic review synthesizes available data on *Salmonella* spp., Shiga toxin‐producing *Escherichia coli* (STEC), *Listeria* spp., *Yersinia* spp., and *Campylobacter* spp. in game meat from hunted wild ungulates. Relevant studies published between 2004 and 2024 were identified through structured database searches and screened using predefined criteria. Data on host species, diagnostic methods, prevalence estimates, isolate characterization, and antimicrobial resistance (AMR) were extracted. Pooled prevalence estimates were determined, stratified by detection methods (culture‐based and PCR‐based) and animal groups (suids and ruminants). A total of 46 publications (170 studies; 223 prevalence estimates) were included. Substantial heterogeneity was observed across the studies. PCR‐based methods yielded higher detection rates than culture, though the magnitude of the difference varied between pathogens. Pathogen occurrence was generally higher in meat from suids, except for STEC, which was more prevalent in the game meat of ruminants. Although many strains appeared to have lower pathogenic potential compared with those typically implicated in human disease, the presence of specific virulence‐associated genes, serogroups, and sequence types linked to human infections indicates that game meat may act as a potential source of infection. AMR was reported in a limited number of studies. Overall, this review highlights substantial methodological variability and important data gaps, particularly regarding virulence‐associated genes, genomic characterization, and AMR. More comprehensive studies spanning a broader range of ecological and production contexts are needed to strengthen the evidence base for the microbiological safety of game meat.

## 1 Introduction

Game meat consumption continues to rise worldwide (Hedman et al. 2020; Mukanganwa, Sukmawati, et al. 2025; Ayaz et al. 2026). Among the hunted ungulate species, commonly hunted deer include the white‐tailed deer (*Odocoileus virginianus*) in North America (Goguen and Riley 2020; Hedman et al. 2020), fallow *(Dama dama*) and red deer (*Cervus elaphus*) in Australia and New Zealand (Forsyth et al. 2023), sika deer (*C. nippon*) in Japan (Okuda et al. 2022), and roe (*Capreolus capreolus*) and red deer in Europe (ENETWILD‐consortium et al. 2021; FACE 2025). Other hunted ungulates include the moose (*Alces alces*) and chamois (*Rupicara rupicara/R. pyrenaica*) in Europe, along with African antelopes such as the impala (*Aepyceros melampus*), springbok (*Antidorcas marsupialis*), and greater kudu (*Tragelaphus strepsiceros*) (Ndlovu et al. 2025). Wild boars (*Sus scrofa*) are especially notable for their near‐global distribution across Europe, Asia, and the Americas (Markov et al. 2022; Kartika et al. 2025).

Since ancient times, hunting has been an important activity for obtaining food. Today, hunting remains a significant source of food in many rural and traditional communities (HLPE 2017), but it is also a popular leisure activity globally (Hedman et al. 2020). In recent decades, hunted wild game meat has gained interest due to its culinary value, high nutritional quality, and sustainability (Costa et al. 2016; Corradini et al. 2022; Ndlovu and Lebopa 2026). It is characterized by high‐quality proteins, low fat content, and a favorable fatty acid profile compared with conventional livestock meat (Bureš et al. 2015; Viganò et al. 2019), while also generating lower greenhouse gas emissions than beef production (Fiala et al. 2020). It is often perceived as more ethical and healthier due to the natural living conditions and absence of veterinary drug use (Costa et al. 2016; Marescotti et al. 2020). However, concerns remain regarding animal welfare (Dickson et al. 2009) and, most importantly, food safety (Marescotti et al. 2021; Ranucci et al. 2021; Mukanganwa, Mukuma, et al. 2025). Game meat production differs substantially from conventional systems, with several factors affecting microbiological safety. Warm and rainy weather, heavier animals, and delayed evisceration are associated with increased bacterial contamination of carcasses (Hedman et al. 2020; Ranucci et al. 2021). Indeed, field evisceration and suboptimal transport and storage conditions may increase bacterial contamination (Sauvala et al. 2019; Ayaz et al. 2026). In addition, the involvement of untrained hunters and the frequent absence of official inspection, particularly in informal markets (Hedman et al. 2020; Ranucci et al. 2021; Mukanganwa, Mukuma, et al. 2025) or meat intended for personal consumption (Hedman et al. 2020), further complicate the implementation of adequate hygiene standards. Nevertheless, proper hygiene is essential because wild animals can host foodborne zoonotic pathogens such as *Salmonella* spp., Shiga toxin‐producing *Escherichia coli* (STEC), *Listeria* spp., enteropathogenic *Yersinia* spp., and *Campylobacter* spp. (Hedman et al. 2020; Altissimi et al. 2023). Campylobacteriosis and salmonellosis are among the most reported zoonoses causing gastroenteritis worldwide (EFSA and ECDC 2025; Walter et al. 2025). *L. monocytogenes* may cause septicemia and death in immunocompromised individuals (Quereda et al. 2021), is widespread in the environment, including soil and water, and can persist in food processing environments (Tuytschaever et al. 2023). Among *Yersinia* spp., only *Y. enterocolitica* and *Y. pseudotuberculosis* are regarded as human enteropathogenic species (Fredriksson‐Ahomaa et al. 2018). However, most *Y. enterocolitica* strains isolated from food belong to biotype 1A, which typically carry the *yst*B gene but lack virulence‐associated genes such as the *ail* and *yst*A genes. These strains are therefore regarded as non or very low pathogenic (Terentjeva et al. 2022), although some biotype 1A strains have been associated with human disease. STEC can cause hemorrhagic colitis and hemolytic uremic syndrome (HUS), with disease severity largely depending on the virulence profile of the strain, particularly *stx* gene variants and the presence of the *eae* gene (Karmali et al. 2010).

In addition to pathogen occurrence and characterization, antimicrobial resistance (AMR) has emerged as an important One Health concern. Although wildlife is generally not exposed to antimicrobial treatments to the same extent as food‐producing animals, wild animals can acquire resistant bacteria through environmental exposure (Pătrînjan et al. 2026). Consequently, the occurrence of antimicrobial‐resistant foodborne pathogens in game meat remains relevant from food safety and public health perspectives.

Despite their importance, existing data on these pathogens in the game meat of hunted ungulates are highly fragmented and highly heterogeneous in study design and methodology, limiting comparability. This systematic review therefore aims to synthesize the available global evidence on the occurrence of *Salmonella*, *Listeria*, STEC, *Yersinia*, and *Campylobacter* in the game meat of hunted ungulates, along with estimating prevalence and identifying gaps in current evidence and assessing the pathogenic potential of reported isolates. By integrating worldwide data, this review provides a comprehensive overview of the current state of knowledge and highlights priorities for future research.

## 2 Methods

### 2.1 Search Strategy and Selection Criteria

On January 28, 2025, a literature search was conducted using Google Scholar, PubMed, and Scopus. The search was limited to articles published from 2004 onwards, corresponding to approximately the most recent 20‐year period. This restriction was applied to focus on evidence generated under contemporary hunting practices, meat handling procedures, and pathogen detection methodologies that are most relevant for current food safety assessments. The search string combined target host species, matrix, and pathogen keywords as follows: [deer OR chamois OR moose OR elk OR (wild AND boar) OR (hunted AND ungulate)] AND [meat OR carcass OR carcase OR organ OR offal OR liver] AND [*Salmonella* OR *Campylobacter* OR *Yersinia* OR stec OR *Listeria*] (Appendix Table S1). The search results were exported to Rayyan software (Ouzzani et al. 2016) for screening and duplicate removal using the built‐in deduplication tool. Titles and abstracts were independently screened by two reviewers (MFA and SB), and discrepancies were discussed until consensus was obtained with a third reviewer (IVD). If the abstract lacked sufficient detail to assess eligibility, the record was retained for full‐text screening. Next, six reviewers (MFA, IVD, BFK, IS, JP, MC) screened the full‐text articles for eligibility. The following inclusion criteria were used: (i) studies conducted after 2004, (ii) target pathogens, that is, *Salmonella* spp., *Campylobacter* spp., *Yersinia* spp., STEC, or *Listeria* spp., (iii) samples from carcasses, cut meat and meat products, or edible offal, and (iv) hunted wild animals belonging to the suids or ruminants. The exclusion criteria were: (i) studies on non‐target pathogens, (ii) samples from blood, tonsils, or feces, (iii) review papers; (iv) studies involving farmed or captive animals; and (v) duplicate records.

Following the initial screening and characterization of eligible studies, we observed that most included publications originated from Europe and predominantly involved species commonly hunted in European settings. To assess whether relevant studies from other geographical regions may have been missed because of species‐specific terminology, a supplementary search was performed using additional host‐species terms representing hunted ungulates and game meat sources commonly reported outside Europe. The review question, databases, pathogen terms, matrix terms, screening procedures, and eligibility criteria remained unchanged. The supplementary search used the following terms for host species: (antelope OR muntjac OR impala OR springbok OR wildebeest OR duiker OR reindeer OR buffalo OR bison OR ibex OR caribou OR (feral AND (hog OR pig)) OR bushmeat). This supplementary search was performed using the same approach as the initial search. All records retrieved through the additional search were screened and assessed using the same predefined eligibility criteria as applied in the primary search to ensure methodological consistency.

**FIGURE 1:**
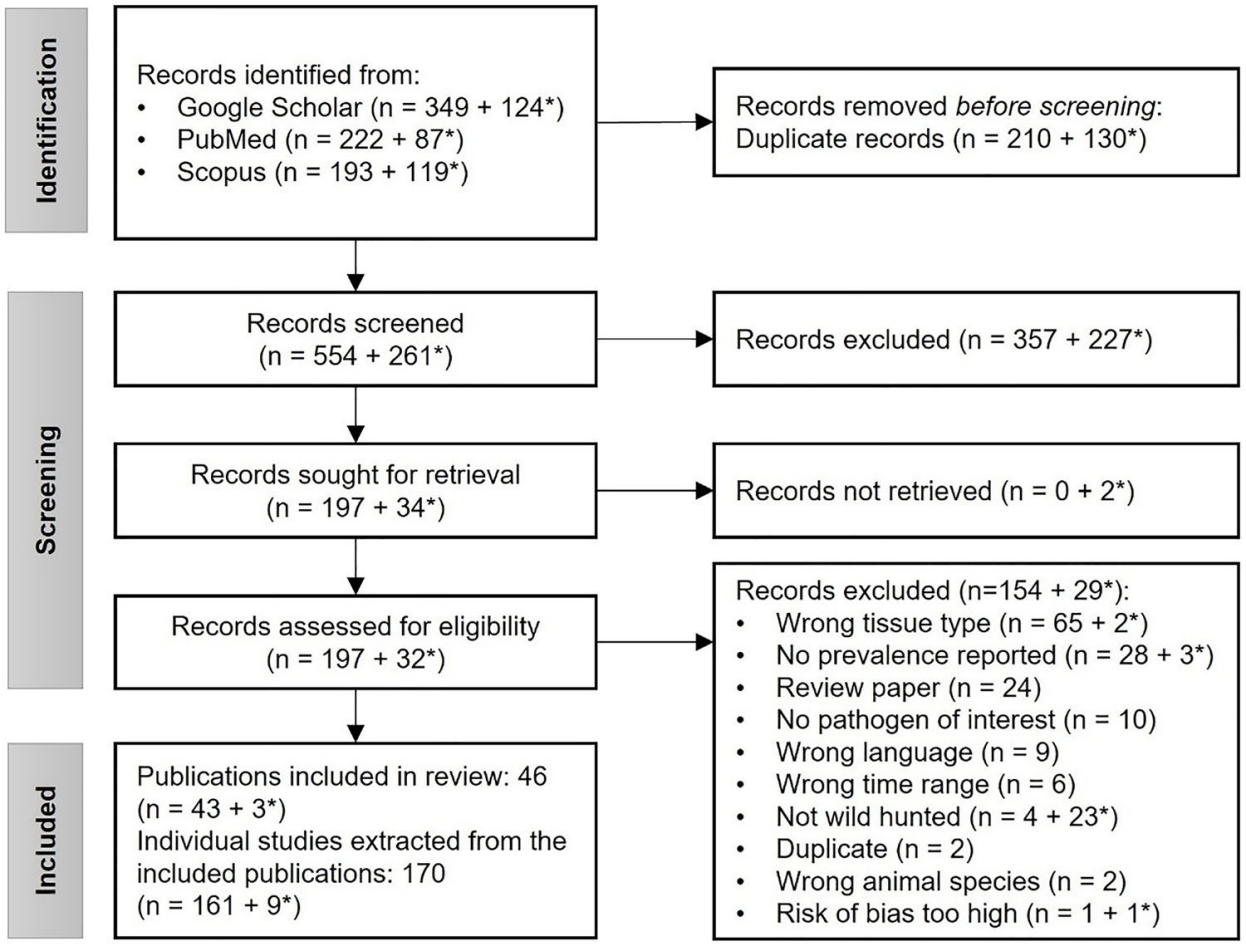
PRISMA flow diagram for study selection. An additional search was carried out, using different host species. Records that were included/excluded during the additional search are indicated with asterisk (*).

The study selection process for both the initial and additional searches, including the number of records identified, screened, excluded (with reasons), and included in the final synthesis, followed the PRISMA 2020 guidelines (Page et al. 2021) and is summarized in the PRISMA flow diagram (Figure 1).

### 2.2 Data Extraction and Analysis

The initial data extraction was performed by five reviewers (BFK, IS, JP, MC, MFA). Each publication was assigned to one reviewer. A data extraction form was developed in Excel to capture information from the articles that met the inclusion criteria after full‐text screening. The following data were extracted: bibliographic details (first author; year of publication), study context (country where the study was conducted), population characteristics (animal species), sample information (matrix, i.e., carcass, raw meat, processed meat, edible offal, or other; sample type, i.e., destructive or swab), pathogen data (genus, species, serotype, virulence genes, sequence types, AMR), detection method (culture or/and molecular), outcomes (number of samples tested, number of positive samples, prevalence estimates). As individual publications could report data for multiple pathogens, animal species, matrices, or detection methods, each unique combination was recorded as a separate observation in the database. These observations are hereafter referred to as “studies” throughout this manuscript. For the analysis, animal species were grouped into two taxonomic categories: suids and ruminant species.

To reduce errors in data collection, the final data extraction of the main outcome variables was performed independently by two reviewers per publication using a standardized extraction form. Pre‐filled contextual variables from the initial database were provided for each study to support the process. For each study, the following variables were extracted: number of tested samples, number of positive samples using molecular methods, and number of positive samples using culture methods. Four reviewers (MFA, BFK, JP, IS) were randomly assigned to the publications using a block design, and each paper was reviewed by two independent reviewers, blinded to each other's results. Discrepancies between data were adjudicated by a third reviewer (IVD).

Bias risk was assessed by one reviewer (JP) using the Joanna Biggs Institute Critical Appraisal Checklist for Prevalence Studies (Munn et al. 2015) using eight questions, covering sampling frame, sampling method, sample size adequacy, description of study subjects and setting, coverage of the identified sample, validity of pathogen identification methods, consistency of measurement, and appropriateness of statistical analysis. The results for each appraisal domain are provided in Appendix Table S2. Each study was scored, and those with a score lower than four were re‐evaluated by the review team. Inclusion of these studies was decided collectively on a case‐by‐case basis, considering the methodological limitations and their potential impact on the review.

### 2.3 Statistical Analysis

All analyses and visualizations were performed using R version 4.4.2. For each pathogen, prevalence data were modeled separately using a generalized linear mixed‐effects model (GLMM) with a binomial distribution and logit link. The outcome variable was the number of positive samples out of the total tested. Studies reporting zero positive samples were retained in all analyses. The detection method (culture vs. PCR), animal group (ruminants vs. suids), and their interactions were included as fixed effects. Considerable heterogeneity was evident across studies, reflected by the wide variation in prevalence estimates, differences in study populations, sampling matrices, geographical regions, and diagnostic methodologies. To account for part of this heterogeneity, publication‐level random effects were included in the models. To account for non‐independence among observations originating from the same publication, publication was included as a random intercept. Alternative random‐effects structures were explored during model development, including nested study‐level effects and publication‐level random slopes; however, these more complex models frequently resulted in singular fits and did not improve model fit. Therefore, the more parsimonious model was retained. Model diagnostics were evaluated using simulation‐based residual analyses implemented in the DHARMa package, and no evidence of substantial overdispersion was detected. All models were fitted using maximum likelihood with Laplace approximation, as implemented in the glmer function from the lme4 package (Bates et al. 2015). For each fitted model, estimated marginal means were obtained using the emmeans package (Lenth 2025). Estimated marginal means were calculated on the response (probability) scale and reported by detection method (culture and PCR) within each animal group (suids and ruminants) to derive the pooled prevalence with corresponding 95% confidence intervals. Contrasts for each fixed effect (detection method and animal group) were calculated marginalized over the levels of the other effect. In addition, all pairwise comparisons across combinations of method and animal groups were performed using the default Tukey adjustment for multiple testing.

## 3 Results

### 3.1 Search and Selection Process

Our initial literature search retrieved 764 articles from three databases (Figure 1). After removal of 210 duplicates, the titles and abstracts of 554 articles were screened for eligibility by two independent reviewers. After consensus discussion with a third reviewer regarding 35 conflicts, a total of 357 records that did not meet the inclusion criteria were excluded. The remaining 197 records underwent full‐text review, of which 154 were excluded, mostly because of the wrong sample type (*n* = 65), not reporting a prevalence outcome (*n* = 28), or because they were review papers (*n* = 24) (Figure 1).

The second search, using additional host species, retrieved 330 records, 32 of which were assessed for eligibility (Figure 1). Most publications were excluded because the animals were not wild and/or hunted (*n* = 23). Three publications from the additional search were included in the review (Bachand et al. 2012; Haindongo et al. 2018; Nkosi et al. 2022), and nine individual studies were extracted from these three publications.

### 3.2 Study Characteristics

In total, 46 publications met the inclusion criteria (Appendix Table S3). They were carried out in 23 countries, mostly in Europe (*n* = 37), of which the vast majority were performed in Italy (*n* = 21). *Salmonella* was the most frequently studied pathogen (*n* = 34 publications), followed by *Listeria* (*n* = 15), STEC (n = 15), *Yersinia* (*n* = 14), and *Campylobacter* (*n* = 11). Several publications reported results for more than one pathogen (*n* = 23). Animal species were classified as suids or ruminants, resulting in 23 publications on suids, eight on ruminants, and 15 publications including both groups. Out of the 38 publications reporting data on meat from wild suids, most studied meat from wild boars (*n* = 36), and only one each from warthogs (*Phacochoerus aethiopicus*) (Kabwang a Mpalang et al. 2013) and red river hogs (*Potamochoerus porcus*) (Bachand et al. 2012). For ruminants, the most frequently studied meat was from red deer (*n* = 11), followed by roe deer (*n* = 9), chamois (*n* = 6), and white‐tailed deer (*n* = 3). *Salmonella*, *Yersinia*, and *Campylobacter* were more frequently studied from the meat of hunted suids than from ruminants, whereas STEC and *Listeria* were reported in the meat of hunted ruminants and suids at similar frequencies (Appendix Table S4).

Carcasses were the most studied game meat matrix (*n* = 22 publications), followed by cut meat and meat products (*n* = 15 publications) and edible offal (*n* = 12 publications) (Appendix Table S5). Cut meat and meat products included raw meat cuts, ground/minced meat, and dried and smoked meat products. *Campylobacter*, *Salmonella*, *Yersinia*, and *Listeria* were studied most often from carcass meat samples. STEC was most frequently studied from cut meat and meat products. Liver was by far the most frequently studied edible offal, being included in 11 out of 12 publications reporting offal samples. In three publications, multiple offal types were examined, namely liver and spleen (Cilia et al. 2021), liver, lungs and spleen (Conedera et al. 2014), and spleen and kidneys (Fredriksson‐Ahomaa et al. 2020).

The prevalence of foodborne bacteria in game meat had been studied using culture‐based detection methods involving enrichment and selective isolation procedures (*n* = 30 publications), PCR detection combined with isolation (*n* = 13), PCR detection only (*n* = 7), or enzyme‐linked fluorescent immunoassay (ELFA) screening followed by isolation (*n* = 2). For *Salmonella*, *Listeria*, and *Yersinia*, culturing with isolation was the most frequently used method (Appendix Table S6). For STEC detection, PCR screening followed by isolation was mostly used. For *Campylobacter*, both culturing and PCR‐based methods were used with similar frequency.

To evaluate the bias risk, each publication was scored across eight domains. Of the 46 included publications, the majority had a score of 8 (*n* = 20) or 7 (*n* = 11), while 7, 5, 2, and 1 publications had a lower score of 6, 5, 4, and 3, respectively.

### 3.3 Pathogen Prevalence in Hunted Game Meat

**FIGURE 2:**
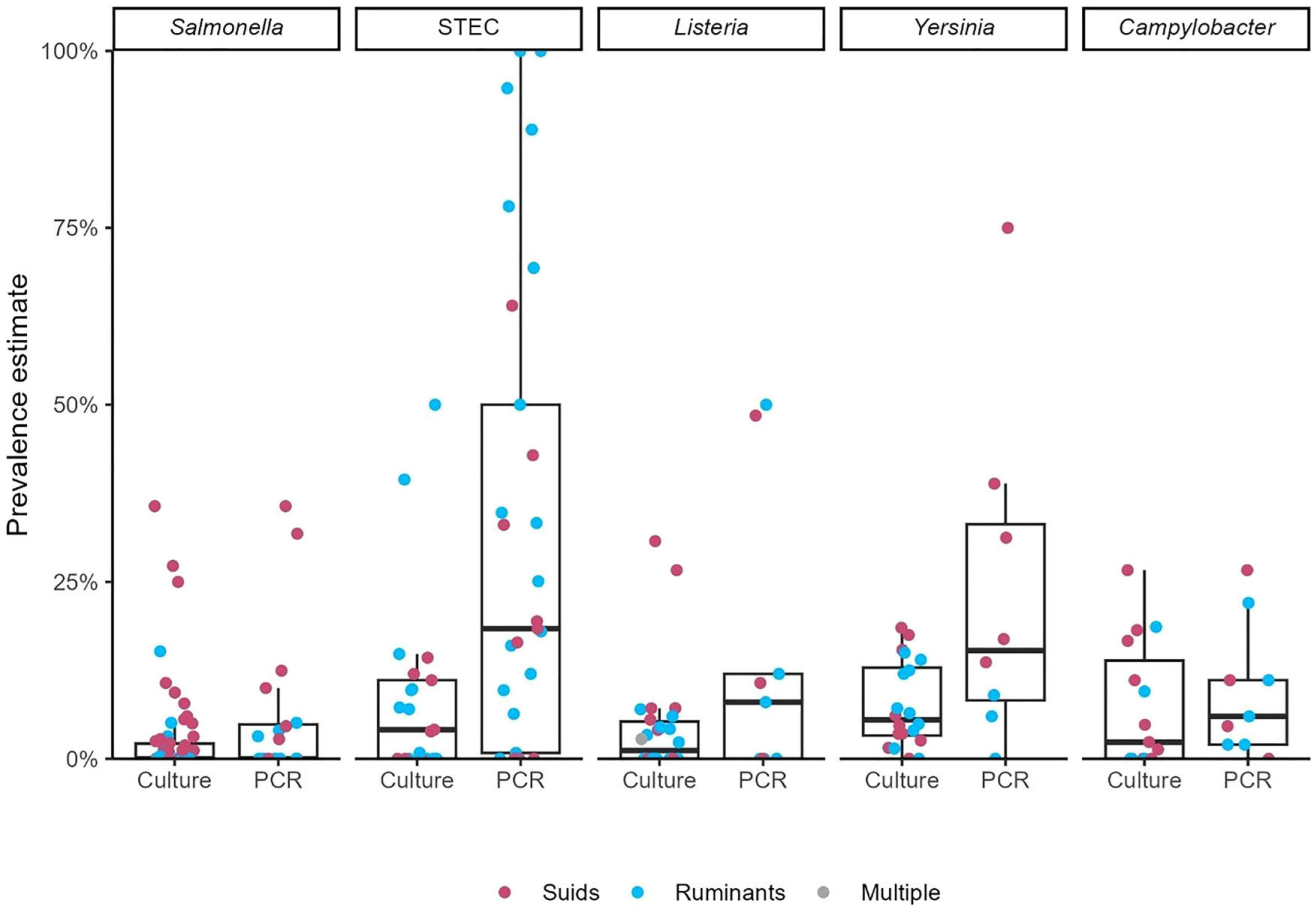
Reported prevalence estimates in meat of hunted ungulates (based on 170 studies extracted from 46 publications), stratified by detection method (culture or PCR‐based). Colors represent the animal category (pink = meat from wild suids, blue = meat from wild ruminants, grey = combination of suids and ruminants).

**FIGURE 3:**
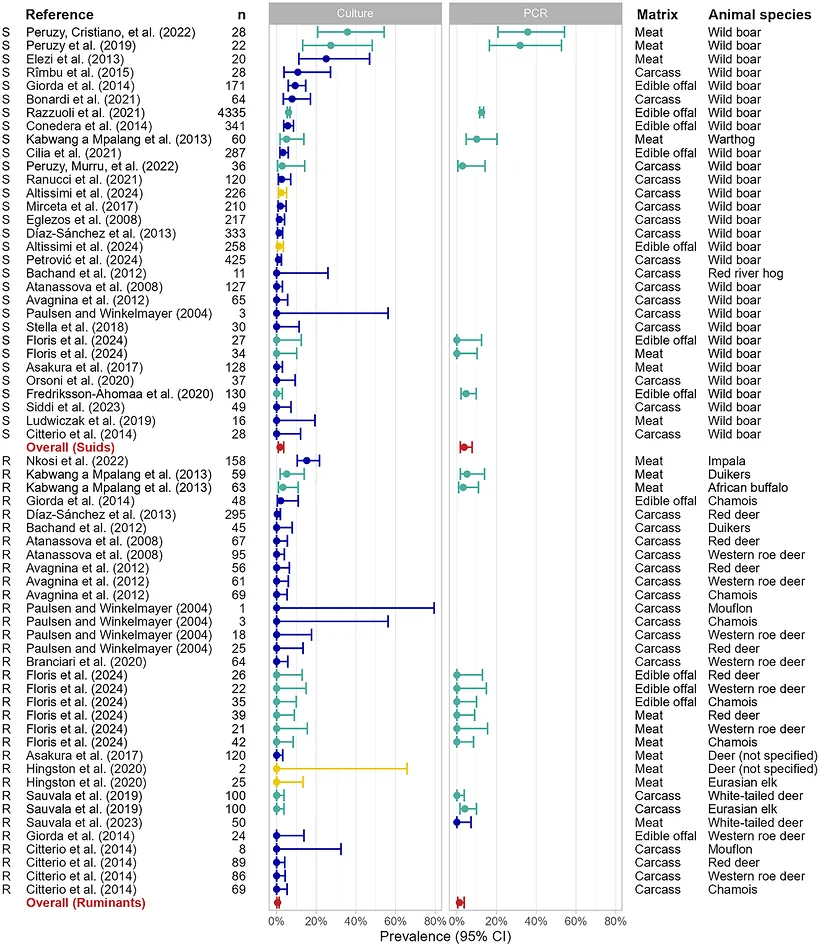
Forest plot of *Salmonella* prevalence estimates from 64 studies extracted from 34 publications, stratified by detection method (culture‐based and PCR) and animal group (S, Suids and R, Ruminants). Prevalence estimates and 95% confidence intervals (CIs) are shown in blue when either culture or PCR was used, in yellow when immunoassay screening was used, and in green when both culture and PCR were applied. Pooled prevalence estimates per animal group are included in red. Study metadata include the publication reference, number of samples (*n*), sample matrix, and animal species. References in Figure 3: (Altissimi et al. 2024; Asakura et al. 2017; Atanassova et al. 2008; Avagnina et al. 2012; Bachand et al. 2012; Bonardi et al. 2021; Branciari et al. 2020; Cilia et al. 2021; Citterio et al. 2014; Conedera et al. 2014; Díaz‐Sánchez et al. 2013; Eglezos et al. 2008; Elezi et al. 2013; Floris et al. 2024; Fredriksson‐Ahomaa et al. 2020; Giorda et al. 2014; Hingston et al. 2020; Kabwang a Mpalang et al. 2013; Ludwiczak et al. 2019; Mirceta et al. 2017; Nkosi et al. 2022; Orsoni et al. 2020; Paulsen and Winkelmayer 2004; Peruzy, Cristiano, et al. 2022; Peruzy et al. 2019; Peruzy, Murru, et al. 2022; Petrovic et al. 2024; Ranucci et al. 2021; Razzuoli et al. 2021; Rîmbu et al. 2015; Sauvala et al. 2019, 2023; Siddi et al. 2024; Stella et al. 2018).

**FIGURE 4:**
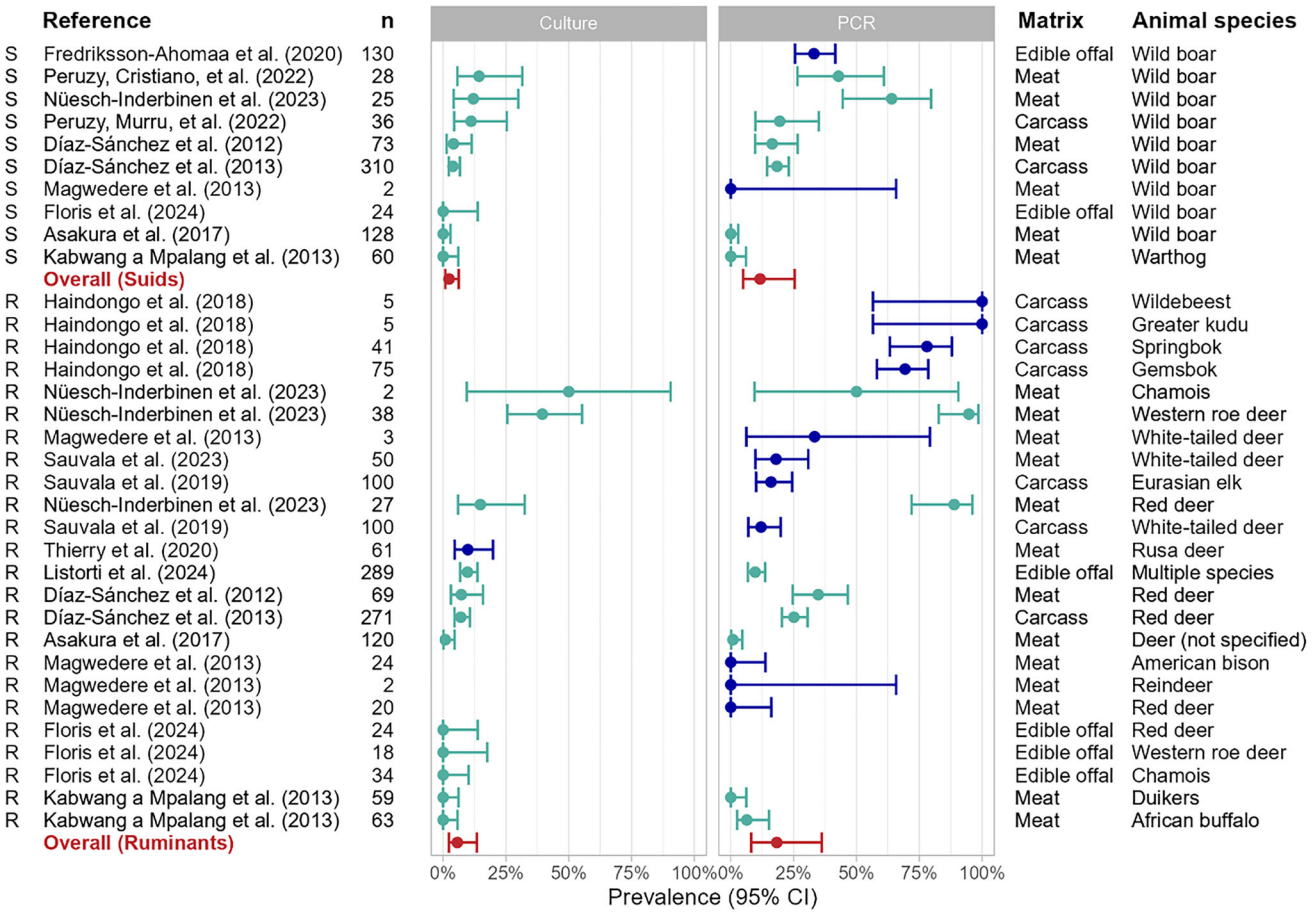
Forest plot of STEC prevalence estimates from 34 studies extracted from 15 publications, stratified by detection method (culture‐based and PCR) and animal group (S, Suids and R, Ruminants). Prevalence estimates and 95% confidence intervals are shown in blue when either culture or PCR was used, and in green when both culture and PCR were applied. Pooled prevalence estimates per animal group are included in red. Study metadata include the publication reference, number of samples (*n*), sample matrix, and animal species. References in Figure 4: (Asakura et al. 2017; Díaz‐Sánchez et al. 2012, Díaz‐Sánchez et al. 2013; Floris et al. 2024; Fredriksson‐Ahomaa et al. 2020; Haindongo et al. 2018; Kabwang a Mpalang et al. 2013; Listorti et al. 2024; Magwedere et al. 2013; Nüesch‐Inderbinen et al. 2023; Peruzy, Cristiano, et al. 2022; Peruzy, Murru, et al. 2022; Sauvala et al. 2019, 2023; Thierry et al. 2020).

**FIGURE 5:**
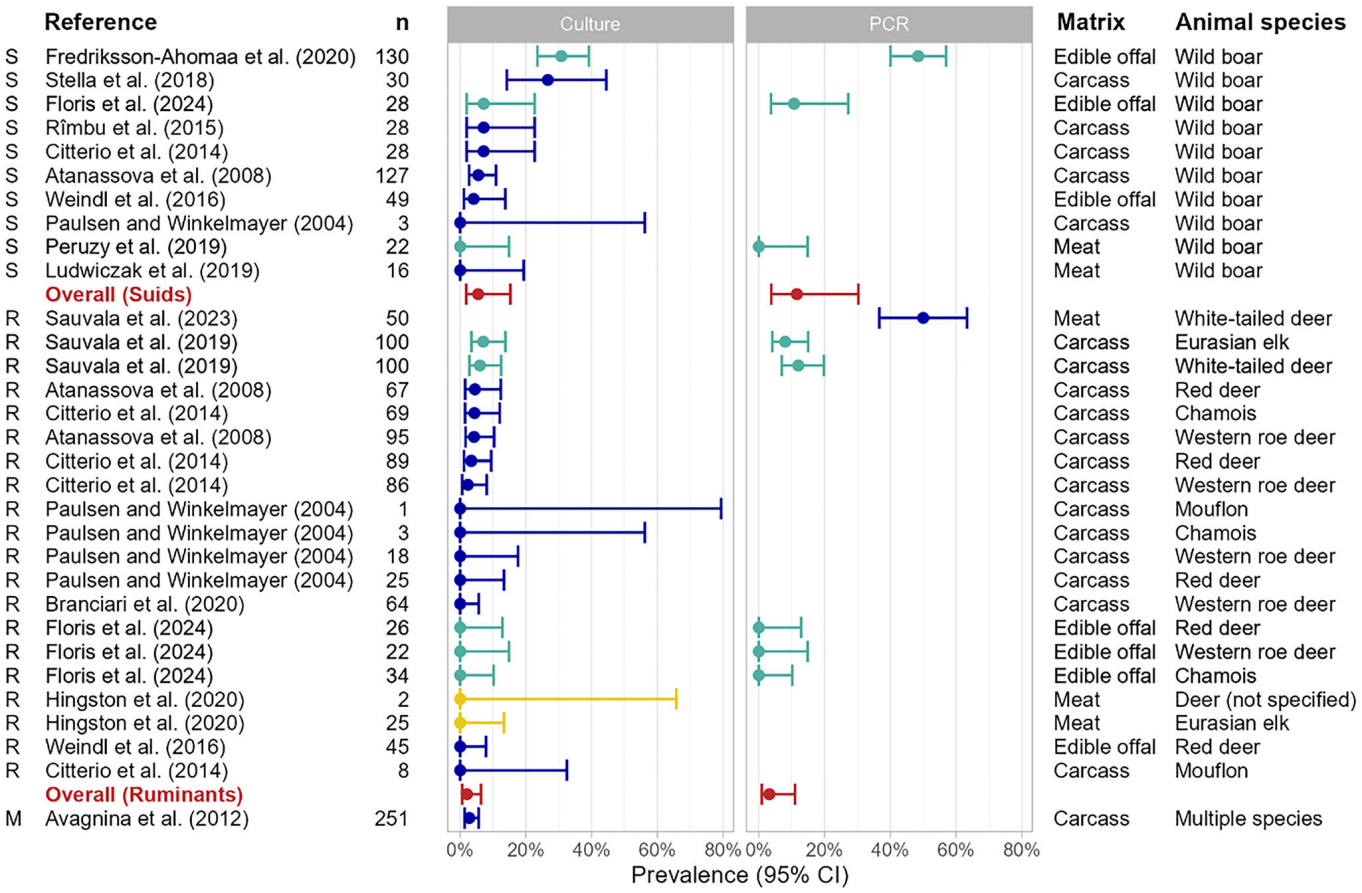
Forest plot of *Listeria* prevalence estimates from 31 studies extracted from 15 publications, stratified by detection method (culture‐based and PCR) and animal group (S, Suids and R, Ruminants). Prevalence estimates and 95% confidence intervals are shown in blue when either culture or PCR was used, in yellow when immunoassay screening was used, and in green when both culture and PCR were applied. Pooled prevalence estimates per animal group are included in red. Study metadata include the publication's reference, number of samples (*n*), sample matrix, and animal species. References in Figure 5: (Atanassova et al. 2008; Avagnina et al. 2012; Branciari et al. 2020; Citterio et al. 2014; Floris et al. 2024; Fredriksson‐Ahomaa et al. 2020; Hingston et al. 2020; Ludwiczak et al. 2019; Paulsen and Winkelmayer 2004; Peruzy et al. 2019; Rîmbu et al. 2015; Sauvala et al. 2019, 2023; Stella et al. 2018; Weindl et al. 2016).

**FIGURE 6:**
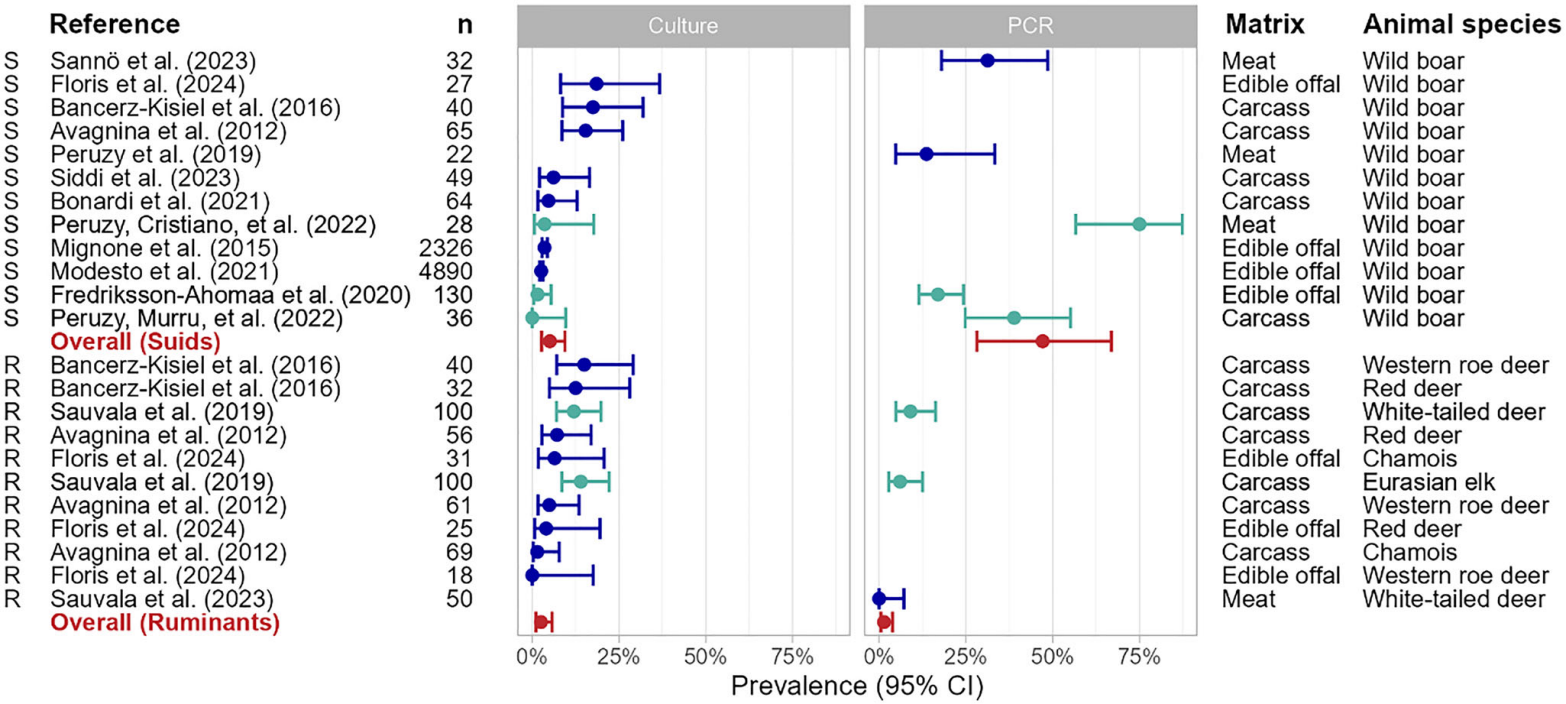
Forest plot of *Yersinia* spp. prevalence estimates from 23 studies extracted from 14 publications, stratified by detection method (culture‐based and PCR) and animal group (S, Suids and R, Ruminants). Prevalence estimates and 95% confidence intervals are shown in blue when either culture or PCR was used, and in green when both culture and PCR were applied. Pooled prevalence estimates per animal group are included in red. Study metadata include the publication's reference, number of samples (*n*), sample matrix, and animal species. References in Figure 6: (Avagnina et al. 2012; Bancerz‐Kisiel et al. 2016; Bonardi et al. 2021; Floris et al. 2024; Fredriksson‐Ahomaa et al. 2020; Mignone et al. 2015; Modesto et al. 2021; Peruzy, Cristiano, et al. 2022; Peruzy et al. 2019; Peruzy, Murru, et al. 2022; Sannö et al. 2023; Siddi et al. 2024).

**FIGURE 7:**
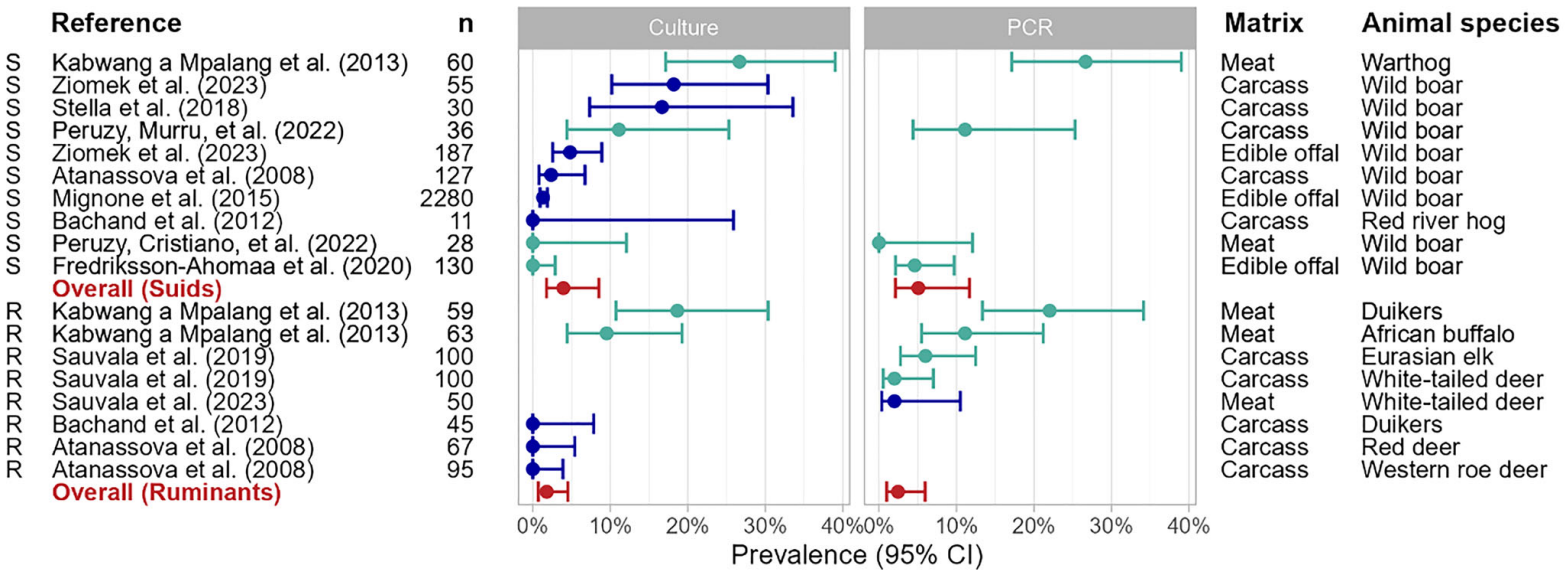
Forest plot of *Campylobacter* prevalence estimates from 18 studies extracted from 10 publications, stratified by detection method (culture‐based and PCR) and animal group (S, Suids and R, Ruminants). Prevalence estimates and 95% confidence intervals are shown in blue when either culture or PCR was used, and in green when both culture and PCR were applied. Pooled prevalence estimates per animal group are included in red. Study metadata include the publication reference, number of samples (*n*), sample matrix, and animal species. References in Figure 7: (Atanassova et al. 2008; Bachand et al. 2012; Fredriksson‐Ahomaa et al. 2020; Kabwang a Mpalang et al. 2013; Mignone et al. 2015; Peruzy, Cristiano, et al. 2022; Peruzy, Murru, et al. 2022; Sauvala et al. 2019, 2023; Stella et al. 2018; Ziomek et al. 2023).

Across the 46 included publications, 170 studies were extracted. In total, 9851 samples were tested for *Salmonella* (*n* = 64 studies), 2316 samples for STEC (*n* = 34 studies), 1641 samples for *Listeria* (*n* = 31 studies), 8291 samples for *Yersinia* (*n* = 23 studies), and 3523 samples for *Campylobacter* (*n* = 18 studies). Each study contributed to one or two prevalence estimates, depending on whether only one detection method was used or whether PCR detection was combined with culturing, yielding a total of 223 prevalence estimates (Figure 2). Overall, pathogen prevalence varied widely between the studies (range 0%–100%), reflecting differences in animal species, matrices, number of samples, and detection methods. Detailed prevalence estimates for individual studies by pathogen are visualized in forest plots in Figures 3, 4, 5, 6, 7 and Appendix Tables S7–S11.

#### 3.3.1 *Salmonella* spp

A total of 82 *Salmonella* prevalence estimates were included in the quantitative analysis, corresponding to 64 unique studies originating from 34 publications (Figure 3). Most publications only used culture methods to detect *Salmonella* (*n* = 23). Six publications used PCR screening followed by culture (Peruzy et al. 2019; Fredriksson‐Ahomaa et al. 2020; Razzuoli et al. 2021; Peruzy, Cristiano, et al. 2022; Peruzy, Murru, et al. 2022; Floris et al. 2024), and two publications used VIDAS immunoassay screening (Hingston et al. 2020; Altissimi et al. 2024) after which culturing was performed on screening‐positive samples. PCR detection and culturing were used in parallel in two publications (Kabwang a Mpalang et al. 2013; Sauvala et al. 2019). One publication used PCR detection only without culture (Sauvala et al. 2023).

In the game meat of suids, 13 out of 31 culture‐based studies reported no *Salmonella* positive samples, whereas prevalence among positive studies ranged from 1 to 36%. For PCR‐based studies (*n* = 8), two studies reported no positive samples, and prevalence among positive studies ranged from 3% to 36%. Among the game meat of ruminants, 27 out of 32 culture‐based studies and 8 out of 11 PCR‐based studies reported no positive samples, with prevalence estimates among positive studies ranging from 0.3% to 15% and from 3% to 5%, respectively. In suid game meat, the pooled prevalence estimates were 1.7% (95% CI 0.8%–3.6%) based on culture methods and 3.7% (1.7%–7.6%) based on PCR results. The pooled prevalence in ruminant game meat was 0.5% (0.2%–1.3%) using culture methods and 1.3% (0.4%–3.7%) using PCR‐based results. The odds of having a positive result were significantly higher using PCR than using culture methods (OR = 2.4, *p* = 0.001) and in suid meat than in ruminant meat (OR = 3.3, *p* < 0.001).

The highest *Salmonella* prevalence rates (32% and 36%) were obtained in wild boar meat from southern Italy by PCR (Peruzy et al. 2019; Peruzy, Cristiano, et al. 2022). Culture confirmation of PCR‐positive samples was high, resulting in similarly elevated prevalence estimates using culture methods (27% and 36%, respectively). A high prevalence (25%) by culturing was also reported in wild boar meat from Albania (Elezi et al. 2013). *Salmonella* prevalence estimates in the meat of wild ruminants were generally low (0%–5%) with both PCR and culturing, except for Nkosi et al. (2022), who reported a culture‐based prevalence of 15% in impala meat around shot wounds in South Africa.

*S. enterica* subspecies and serotypes were reported in 14 publications. However, characterization of isolates from ruminant game meat was limited because *Salmonella* was only rarely isolated from these samples. Consequently, only two publications reported characterized isolates from ruminant meat: *S. enterica* subspecies *houtenae* was isolated from a chamois liver in Italy (Giorda et al. 2014) and *S. enterica* subspecies *enterica* serotype Corvalis from a deer carcass in Spain (Díaz‐Sánchez et al. 2013). In contrast, more extensive characterization results were available for isolates from suid game meat. Razzuoli et al. (2021) identified all six subspecies (*arizonae, diarizonae, enterica, houtenae, indica*, and *salamae*) in suid livers, of which subspecies *enterica* was the most common, followed by *salamae* and *diarizonae*. In total, 30 serotypes of the subspecies *enterica* were identified in suid meat, as reported in 13 publications (Table 1). The most common serotypes were Typhimurium, Stanleyville, and Enteritidis, which were reported by ten, four, and three publications, respectively.

**TABLE 1:** *Salmonella enterica* subspecies *enterica* serotypes in wild boar meat reported in 13 publications.

*N*	Serotypes (*N*)	Country	Reference
1	Typhimurium (1)	Albania	Elezi et al. (2013)
3	Kottbus (1), Lansing (1), Potsdam (1)	Australia	Eglezos et al. (2008)
8	Stanleyville (6), Derby (1), Typhimurium (1)	Italy	Altissimi et al. (2024)
4	Enteritidis (2), Agama (1), Typhimurium (1)	Italy	Bonardi et al. (2021)
5	Newport (2), Infantis (1), Kottbus (1), Rubislaw (1)	Italy	Cilia et al. (2021)
19	Cholerasuis (17), Typhimurium (1), Not identified (1)	Italy	Conedera et al. (2014)
7	Typhimurium (4), Stoneferry (1), Veneziana (1), Ried (1)	Italy	Giorda et al. (2014)
6	Kasenyi (3), Stanleyville (2), Typhmurium (1)	Italy	Peruzy et al. (2019)
10	Kasenyi (4), Thompson (2), Coeln (1), Manhattan (1), Stanleyville (1), Veneziana (1)	Italy	Peruzy, Cristiano, et al. (2022)
3	Stanleyville (2), Typhimurium (1)	Italy	Ranucci et al. (2021)
157	Napoli (21), Enteritidis (20), Typhimurium (14), Veneziana (10), Coeln (9), Canada (8), Newport (8), Thompson (8), Oxford (7), Kimuenza (6), Munster (6), Kottbus (5), Banjul (4), Galil (4), Brandenburg (3), Infantis (3), Stourbridge (3), and others	Italy	Razzuoli et al. (2021)
5	Enteritidis (4), Typhimurium (1)	Serbia	Petrovic et al. (2024)
3	Bardo (1), Montevideo (1), Typhimurium (1)	Spain	Díaz‐Sánchez et al. (2013)

*Note: N* = number of isolates.

#### 3.3.2 STEC

The reported STEC prevalence estimates ranged from 0% to 100% across 50 extracted prevalence estimates corresponding to 34 unique studies, extracted from 15 publications (Figure 4). The STEC prevalence estimates of one publication were excluded because sample screening was based on the VIDAS immunoassay targeting only serotype O157:H7 without screening the *stx* genes (Hingston et al. 2020). In ruminant game meat, 5 out of 13 culture‐based studies and 4 out of 20 PCR‐based studies reported no STEC‐positive samples. Among studies reporting positive samples, prevalence ranged from 0.8% to 50% and 0.8% to 100%. In suid game meat, 3 of 8 culture‐based studies and 3 of 9 PCR‐based studies reported no positives, whereas prevalence estimates among positive studies ranged from 4% to 14% and 16% to 64%, respectively. Model‐based pooled estimates indicated a higher prevalence in ruminant meat (culture 5.6% [2.3%–13.4%]; PCR 18.3% [8.1%–36.2%]) than in suid meat (culture 2.4% [0.9%–6.2%]; PCR 11.6% [4.9%–25.4%]). The odds of finding a positive sample were significantly higher when using PCR than culture (OR = 4.5; *p* < 0.001) and lower in the meat of suids than that of ruminants (OR = 0.5; *p* < 0.001).

Concerning STEC detection, the methods applied varied greatly among the studies, which likely contributed to the large variations in the outcomes. Most publications (9 out of 15) used PCR screening followed by culture, five used PCR detection without culture, and one only used culture methods (Figure 4).

Nine studies combined a PCR screening step followed by the culturing of presumptive‐positive samples targeting *stx1*/*stx2* (Díaz‐Sánchez et al. 2012, Díaz‐Sánchez et al. 2013; Asakura et al. 2017; Nüesch‐Inderbinen et al. 2023) or *stx1*/*stx2* and *eae* (Kabwang a Mpalang et al. 2013; Peruzy, Cristiano, et al. 2022; Peruzy, Murru, et al. 2022; Floris et al. 2024; Listorti et al. 2024). Isolation rates within these studies generally ranged from 0% in the smoked meat samples of common duiker (*Sylvicapra grimmia*), African buffalo (*Syncerus caffer*), and desert warthog (*P. aethiopicus*) in the Democratic Republic of the Congo (DRC), as well as in livers of chamois, deer, and wild boars in Italy, to 10% in various deer and wild boar meat samples. However, the highest isolation rates were reported by Nüesch‐Inderbinen et al. (2023) in game meat from roe deer (39%), red deer (15%), and wild boar (12%) originating from multiple European countries.

Five publications relied solely on the PCR detection of enriched samples. These publications targeted either *stx1*/*stx2* (Fredriksson‐Ahomaa et al. 2020; Sauvala et al. 2019, 2023) or *stx1*/*stx2* in combination with *eae* (Magwedere et al. 2013; Haindongo et al. 2018). Within these publications, presumptive STEC prevalence estimates ranged from 0% in the meat of American bison (*Bison bison*), red deer, and reindeer in the United States, to 100% in the carcasses of wildebeest (*Chonnocaetes taurinus*) and greater kudu in Namibia (Magwedere et al. 2013; Haindongo et al. 2018), though the latter results should be interpreted with caution due to the limited sample size (*n* = 5 each). Nevertheless, the same authors also found high prevalence estimates from the carcasses of springbok (78%, *n* = 41) and gemsbok (*Oryx gazella*) (69%, *n* = 75).

Thierry et al. (2020) used a culture method without prior PCR screening to isolate STEC from rusa deer (*C. timorensis*) meat samples from Mauritius, using a chromogenic STEC agar (CHROMagar STEC) after non‐selective enrichment, followed by PCR confirmation of colonies targeting *stx1*, *stx2*, and *eae*. STEC carrying *stx*2 was isolated from 5 out of 61 (8%) rusa deer meat samples.

In six out of ten publications using culture methods, 102 STEC isolates were characterized, including 89 isolates from ruminant meat and 13 from suid meat (Table 2). Subtypes of *stx* were reported in four publications, and multi‐locus sequence types (MLST) in two publications. The O serogroup could not be identified in 33% (33/102) of the STEC isolates (Table 2). Serogroup O146 predominated, being identified in 27% (24/89) of ruminant isolates (Díaz‐Sánchez et al. 2013; Thierry et al. 2020; Nüesch‐Inderbinen et al. 2023; Listorti et al. 2024) and 23% (3/13) of suid isolates (Díaz‐Sánchez et al. 2013). Serogroup O27 was also frequently identified in ruminants (11/89; 12%) (Díaz‐Sánchez et al. 2012, 2013; Nüesch‐Inderbinen et al. 2023). Both *stx*1 and *stx*2 were found, but *stx*2 was predominant (82/102; 80%), occurring alone (71/102; 70%) or in combination with *stx*1 (14/102; 14%). In four publications, s*tx* gene subtyping was performed on 84 isolates. The most common subtype was *stx*2b (46/84; 55%), mainly alone (38/46; 83%) or combined with *stx*1c (5/46; 11%) or *stx*1d (3/46; 6.5%). Other subtypes (s*tx*1c, *stx*1d, *stx*2a, *stx*2e, and *stx*2g) were less frequent (1%–15%; Table 2). The *eae* gene was detected in 12% (12/102) of STEC isolates, exclusively found in the livers of chamois (3 isolates carrying *stx*2b) and roe deer (9 isolates carrying *stx*1a, *stx*1d, *stx*2b, and/or *stx*2) in Italy (Listorti et al. 2024).

**TABLE 2:** Characteristics of STEC isolates reported in 14 studies extracted from six publications.

Animal species	*N*	*stx or stx* subtypes (*N*)	*eae* (*N*)	Serogroup/Serotype (*N*)	MLST (*N*)	Country	Reference
Chamois	8	*stx2b* (8)	*eae* (3)	O146 (1), NI (7)	NS	Italy	Listorti et al. (2024)
	1	*stx1c *+ *stx2b* (1)	*eae* (0)	O76:H19 (1)	ST675 (1)	Switzerland	Nüesch‐Inderbinen et al. (2023)
Fallow deer	8	*stx1d* (2), *stx1d *+ *stx2b* (2), *stx2b* (2), *stx1c* (1), *stx1c *+ *stx2d* (1)	*eae* (0)	O113 (2), O145 (1), O146 (1), NI (4)	NS	Italy	Listorti et al. (2024)
Red deer	5	*stx2b* (3), *stx2e* (1), *stx2g* (1)	*eae* (0)	O27:H30 (3), O8:H9 (1), O187:H28 (1)	ST753 (3), ST23 (1), ST200 (1)	Austria, Hungary, Slovenia, Switzerland	Nüesch‐Inderbinen et al. (2023)
	2	*stx2g* (2)	*eae* (0)	NI (2)	NS	Italy	Listorti et al. (2024)
	5	*stx2b* (4), *stx2a* (1)	*eae* (0)	O27:H30 (4), O11:H5 (1)	NS	Spain	Díaz‐Sánchez et al. (2012)
	10	*stx2* (9), *stx1 *+ *2* (1)	*eae* (0)	O27:H30 (4), O146:H (2), O8:H2 (1), O43:H2 (1), O76:H2 (1), ONT:H21 (1)	NS	Spain	Díaz‐Sánchez et al. (2013)
Roe deer	18	*stx2b* (10), *stx1c* (5), *stx1c *+ *stx2b* (3)	*eae* (0)	O146:H28 (8), O110:H31 (5), O21:H21 (3), ONT:H8 (2)	ST738 (8), ST812 (5), ST56 (2), ST26 (2), UN^e^ (1)	Germany, Poland, Switzerland	Nüesch‐Inderbinen et al. (2023)
	26	*stx2b* (8), *stx2* (5), *stx1d* (4), *stx1a *+ *stx2* (3), *stx1a* (2), *stx2g* (2), *stx1c* (1), *stx1d *+ *stx2b* (1)	*eae* (9)	O146 (7), O104 (3), O113 (1), NI (15)	NS	Italy	Listorti et al. (2024)
Rusa deer	5	*stx2* (5)	*eae* (0)	O146 (5)	ST738 (1)	Mauritius	Thierry et al. (2020)
Sika deer	1	*stx1d* (1)	*eae* (0)	ONT:H25 (1)	NS	Japan	Asakura et al. (2017)
Wild boar	3	*stx2b* (1), *stx2g* (1), *stx1c *+ *stx2b* (1)	*eae* (0)	O27:H30 (1), O166:H28 (1), ONT:H31 (1)	NS	Spain	Díaz‐Sánchez et al. (2012)
	7	*stx2* (6), *stx1 *+ *stx2* (1)		O54:H‐ (2), O2:H‐ (1), O130:H38 (1), O146:H‐ (1), O168:H8 (1), O173:H16 (1)	NS	Spain	Díaz‐Sánchez et al. (2013)
	3	*stx2b* (2), *stx2a* (1)	*eae* (0)	O146:H28 (2), O179:H8 (1)	ST738 (2), UN (1)	Switzerland	Nüesch‐Inderbinen et al. (2023)

*Note: N* = number of isolates.

Abbreviations: MLST, multi‐locus sequence type; NI, not identified; NS, not studied; UN, unknown.

Nüesch‐Inderbinen et al. (2023) applied serotyping, virulotyping, and MLST on 27 STEC isolates from 23 game meat samples in Central Europe and revealed that ST738, ST812, and ST753 were the most prevalent (Table 2), identifying ST738 and ST812 in roe deer meat from Germany, Poland, and Switzerland and ST753 in red deer meat from Slovenia and Switzerland. ST738 was also identified in wild boar meat from Switzerland. In particular, STEC O146:H28 ST738 (*n* = 10) harboring *stx*2b were detected in both roe deer and wild boar meat, STEC O110:H31 ST812 (*n* = 5) harboring *st*1c + *stx*1c or *stx*2b were found in roe deer meat and STEC O27:H30 ST 753 (*n* = 3) harboring *stx*2b were isolated from red deer meat in Slovenia and Switzerland. Thierry et al. (2020) sequenced one isolate from rusa meat in Mauritius, which was identified as O146, ST738.

#### 3.3.3 *Listeria* spp.

A total of 39 *Listeria* prevalence estimates were included in the quantitative analysis, corresponding to 31 unique studies originating from 15 publications (Figure 5). Only culture methods were used in most publications (*n* = 9). *L. monocytogenes* was screened with PCR before culturing in three publications (Peruzy et al. 2019; Fredriksson‐Ahomaa et al. 2020; Floris et al. 2024), and PCR detection and culturing were used in parallel in one publication (Sauvala et al. 2019). Immunoassay screening with culture confirmation (*n* = 1) (Hingston et al. 2020) or PCR only (*n* = 1) (Sauvala et al. 2023) were also used. Among studies investigating suid game meat, 3 of 10 culture‐based studies and 1 of 3 PCR‐based studies reported no *Listeria* detection. Positive studies reported prevalence estimates ranging from 4% to 31% and 11% to 48%, respectively. For ruminants, 12 of 19 culture‐based studies and 3 of 6 PCR‐based studies reported no positives, while prevalence among positive studies ranged from 2% to 7% and 8% to 50%, respectively. In the game meat of suids, the pooled estimates were 5.5% (1.8%–15.3%) using culture and 11.6% (3.8%–30.3%) using PCR‐based data. The pooled prevalence in ruminant game meat was 2.0% (0.6%–6.3%) using culture and 3.2% (0.9%–11.0%) using PCR‐based data. The odds of having a positive result were significantly higher in suid meat than in ruminant meat (OR = 3.3, *p* = 0.004). The odds of obtaining a positive result were significantly higher using PCR‐based methods than culture‐based methods (OR = 1.9, *p* = 0.004). The highest prevalence of *L. monocytogenes* (48%) was reported in wild boar offal from Finland using PCR, while the culture of PCR‐positive samples also showed a high prevalence (31%) (Fredriksson‐Ahomaa et al. 2020). A high *L. monocytogenes* prevalence (50%) was also reported in vacuum‐packed raw meat cuts of white‐tailed deer in Finland using PCR only (Sauvala et al. 2023). However, in most studies, the prevalence was below 8% (Figure 5).

*L. monocytogenes* was identified in 11 studies extracted from 7 publications (Avagnina et al. 2012; Citterio et al. 2014; Rîmbu et al. 2015; Weindl et al. 2016; Sauvala et al. 2019; Fredriksson‐Ahomaa et al. 2020; Floris et al. 2024). *L. monocytogenes* serogroups were reported in five studies extracted from four publications (Weindl et al. 2016; Sauvala et al. 2019; Fredriksson‐Ahomaa et al. 2020; Floris et al. 2024). Most of the isolates (97%, 64/66) belonged to serogroup IIa and two isolates to serogroup IVb. Serogroup IVb was identified in *L. monocytogenes* isolated from wild boar offal (Fredriksson‐Ahomaa et al. 2020). In two publications, 15 sequence types were identified out of a total of 62 isolates (Sauvala et al. 2019; Fredriksson‐Ahomaa et al. 2020).

#### 3.3.4 *Yersinia* spp.

A total of 28 *Yersinia* prevalence estimates were included in the quantitative analysis, corresponding to 23 unique studies originating from 14 publications (Figure 6). Half of the publications only used culture methods (*n* = 7), while the others used PCR to screen for positive samples before culturing (*n* = 3) (Fredriksson‐Ahomaa et al. 2020; Peruzy, Cristiano, et al. 2022; Peruzy, Murru, et al. 2022) in parallel with culturing (*n* = 1) (Sauvala et al. 2019) or without culture confirmation (*n* = 3) (Peruzy et al. 2019; Sannö et al. 2023; Sauvala et al. 2023). In the game meat of suids, one of ten culture‐based studies and none of five PCR‐based studies reported no *Yersinia*‐positive samples. Among positive studies, prevalence estimates ranged from 1.5% to 19% and 14% to 75%, respectively. In meat from wild ruminants, 1 of 10 culture‐based studies and 1 of 3 PCR‐based studies reported no positive samples, with prevalence estimates among positive studies ranging from 1.4% to 15% and 6 to 9%, respectively. In the game meat of suids, the pooled estimates were 5.1% (2.7%–9.4%) using culture and 47.1% (28.2%–67.0%) using PCR‐based data. The pooled prevalence in ruminant game meat was 2.5% (95% CI 1.1%–5.7%) using culture and 1.4% (0.4%–3.8%) using PCR‐based data. The odds of having a positive result were significantly higher in suid meat than in ruminant meat (OR = 11.5, *p* < 0.001). The odds of obtaining a positive result were significantly higher using PCR than using culture methods (OR = 3.0, *p* < 0.001). High prevalence rates (14%–75%) were reported in wild boar meat by PCR (Sannö et al. 2018; Peruzy et al. 2019; Fredriksson‐Ahomaa et al. 2020; Peruzy, Cristiano, et al. 2022; Peruzy, Murru, et al. 2022), whereas *Yersinia* prevalence was considerably lower (0%–9%) with PCR in the game meat of white‐tailed deer and moose (Sauvala et al. 2019, Sauvala et al. 2023). *Yersinia* spp. were isolated from game meat in most studies (Figure 6).

In 4 out of 7 publications using PCR detection, the *ail* gene was used as the target to detect pathogenic *Y. enterocolitica* (biotypes 1B, 2–5) and *Y. pseudotuberculosis* (Fredriksson‐Ahomaa et al. 2020; Sannö et al. 2023; Sauvala et al. 2023, Sauvala et al. 2019). A high prevalence (31%) of *ail*‐positive *Y. enterocolitica* was reported in minced wild boar meat in Sweden (Sannö et al. 2023). A clearly lower prevalence of *ail*‐positive *Yersinia* (including both *Y. enterocolitica* and *Y. pseudotuberculosis*) were reported in the meat of white‐tailed deer (9%) and moose (6%) in Finland (Sauvala et al. 2019, 2023). Three publications used the *yst*B and *yst*A genes to detect non‐ or low‐pathogenic (biotype 1A) and pathogenic (biotypes 1B, 2–5) *Y. enterocolitica*, respectively (Peruzy et al. 2019; Peruzy, Cristiano, et al. 2022; Peruzy, Murru, et al. 2022). The prevalence was lower in studies using *yst*A compared with *yst*B. The detection rates using *yst*A varied between 0% and 21%, whereas the detection rates using *yst*B varied between 14% and 75%.

*Y. enterocolitica* was isolated in 16 studies, extracted from nine publications (Table 3). Most of the isolates belonged to biotype 1A and carried the *yst*B gene. In some studies, biotypes 1B and 2 were identified (Mignone et al. 2015; Bancerz‐Kisiel et al. 2016; Modesto et al. 2021). The *ail* and *yst*A virulence genes were reported in some *Y. enterocolitica* studies (Sauvala et al. 2019; Modesto et al. 2021; Floris et al. 2024). Biotype 1A isolates found in wild boar livers from Italy were frequently *ail‐* (44%) or *yst*A‐ (20%) positive (Modesto et al. 2021). *Y. pseudotuberculosis* serotype O1, carrying the *virF* gene on the virulence plasmid, was isolated in one study, from wild boar kidney/spleen samples (Fredriksson‐Ahomaa et al. 2020). All isolates carried both chromosomal and plasmid‐borne virulence genes. In some studies, *Yersinia* isolates were sequenced (Sauvala et al. 2019; Floris et al. 2024; Siddi et al. 2024). Several sequence types were identified among *Y. enterocolitica* isolates. All sequence types were unknown types or types not associated with human infections. *Y. pseudotuberculosis* isolates belonged to sequence type ST42.

**TABLE 3:** Characteristics of *Y. enterocolitica* isolates reported in eight publications.

Animal species	*N*	Biotype (*N*)	Virulence genes				ST (*N*)	Matrix	Country	Reference
			*yst*B	*yst*A	*ail*	*vir*F				
Chamois	1	NS	0	0	0	NS	NS	Carcass	Italy	Avagnina et al. (2012)
	2	1A (2)	2	0	1	NS	UN (2)	Liver	Italy	Floris et al. (2024)
Moose	1	1A (1)	NS	NS	1	NS	UN (1)	Carcass	Finland	Sauvala et al. (2019)
Red deer	1	NS	0	0	0	NS	NS	Carcass	Italy	Avagnina et al. (2012)
	1	1A (1)	1	0	1	NS	UN (1)	Liver	Italy	Floris et al. (2024)
	6	1A (6)	6	0	0	NS	NS	Carcass	Poland	Bancerz‐Kisiel et al. (2016)
Roe deer	1	NS	0	0	0	NS	NS	Carcass	Italy	Avagnina et al. (2012)
	4	1A (4)	4	0	0	NS	NS	Carcass	Poland	Bancerz‐Kisiel et al. (2016)
White‐tailed deer	8	1A (8)	NS	NS	3	NS	UN (3)	Carcass	Finland	Sauvala et al. (2019)
Wild boar	3	NS	0	0	0	NS	NS	Carcass	Italy	Avagnina et al. (2012)
	5	1A (5)	5	0	2	NS	UN (5)	Liver	Italy	Floris et al. (2024)
	82	1A (79), 1B (3)	NS	NS	NS	NS	NS	Liver	Italy	Mignone et al. (2015)
	126	1A (117), 1B (8), 2 (1)	88	25	55	NS	NS	Liver	Italy	Modesto et al. (2021)
	1	1A (1)	1	0	0	NS	NS	Cut meat	Italy	Peruzy, Cristiano, et al. (2022)
	3	1A (3)	1	0	0	0	ST332 (1), ST860 (2)	Carcass	Italy	Siddi et al. (2024)
	8	1A (7), 1B (1)	8	0	0	NS	NS	Carcass	Poland	Bancerz‐Kisiel et al. (2016)

*Note: N* = number of isolates.

Abbreviations: NS, not studied; ST, sequence type; UN, unknown.

#### 3.3.5 *Campylobacter* spp

A total of 24 *Campylobacter* prevalence estimates were included in the quantitative analysis, corresponding to 18 unique studies originating from 11 publications (Figure 7). Five publications used a combination of PCR and culture methods, five used culture methods only, and one used PCR detection only. For suid game meat, 3 of 10 culture‐based studies and 1 of 4 PCR‐based studies reported no *Campylobacter* detection. Among studies reporting at least one positive sample, prevalence estimates ranged between 1% and 27%, and 5% to 27%, respectively. For ruminants, 3 of 5 culture‐based studies and none of five PCR‐based studies reported no positives, with prevalence estimates among positive studies ranging from 9% to 19%, and 2% to 22%, respectively. The pooled prevalence estimates were consistently higher in the meat of hunted suids, that is, 3.9% (95% CI: 1.8%–8.5%) using culture and 5.1% (2.1%–11.7%) using PCR, than in ruminant meat, which reached 1.8% (0.1%–4.5%) using culture and 2.5% (1.0%–6.0%) using PCR. Although PCR‐based methods yielded numerically higher prevalence estimates than culture‐based methods, the difference was not statistically significant (OR = 1.3, *p* = 0.21). Meat from wild suids was associated with higher odds of positive *Campylobacter* findings (OR = 2.1, *p* = 0.002) than meat from ruminants. High *Campylobacter* prevalence estimates were reported in game meat from Central Africa (DRC) (Kabwang a Mpalang et al. 2013). The highest prevalence was found in smoked meat from warthog (26%) with both PCR and culture methods.

In two publications, *Campylobacter* was identified only at the genus level (Mignone et al. 2015; Stella et al. 2018). In four other publications, the species of *Campylobacter* were determined (Table 4). *C. coli* was detected in all studies and was reported as the predominant species in smoked game meat from ruminants and suids in DRC (Kabwang a Mpalang et al. 2013). *C. jejuni* was identified in four publications, while *C. lanienae* was found in wild boar liver and carcass samples in one publication (Ziomek et al. 2023).

**TABLE 4:** *Campylobacter* isolates found in game meat in four publications.

Animal species	*N*	*Campylobacter* species (*N*)	Matrix	Country	Reference
Buffalo	6	*C. coli* (6)	Smoked meat	DRC	Kabwang a Mpalang et al. (2013)
Duiker	11	*C. coli* (8), *C. jejuni* (3)	Smoked meat	DRC	Kabwang a Mpalang et al. (2013)
Warthog	16	*C. coli* (12), *C. jejuni* (4)	Smoked meat	DRC	Kabwang a Mpalang et al. (2013)
Wild boar	3	*C. coli* (2), *C. jejuni* (1)	Carcass	Germany	Atanassova et al. (2008)
	4	*C. coli* (1), *C. jejuni* (1), *Campylobacter* spp. (2)	Carcass	Italy	Peruzy, Murru, et al. (2022)
	9	*C. coli* (3), *C. jejuni* (3), *C. lanienae* (3)	Liver	Italy	Ziomek et al. (2023)
	10	*C. coli* (5), *C. lanienae* (5)	Carcass	Italy	Ziomek et al. (2023)

*Note: N* = number of isolates.

Abbreviation: DRC, Democratic Republic of the Congo.

#### 3.3.6 Antimicrobial Resistance

AMR in foodborne bacteria isolated from game meat was reported in nine publications, which were all performed in Europe. AMR was assessed in isolates of *Salmonella*, STEC, *Listeria*, and *Yersinia* (Table 5). The presence of resistance genes was reported in three publications (Nüesch‐Inderbinen et al. 2023; Floris et al. 2024; Siddi et al. 2024).

**TABLE 5:** Antimicrobial resistance among foodborne bacteria isolated from game meat extracted from nine publications.

Bacterial species	Animal species	*N*	Resistance (*N*; gene)	Method	Country	Reference
*Salmonella* spp.	Wild boar	5	sulfamethoxazole (1)	Phenotypic	Italy	Bonardi et al. (2021)
	Wild boar	11	streptomycin (6), cephalothin (2) MDR (1): tetracycline, enrofloxacin, nitrofurantoin, nalidixic acid, streptomycin	Phenotypic	Italy	Cilia et al. (2021)
	Wild boar	260	MDR (25), sulphonamides (238), sulfamethoxazole‐trimethoprim (57), tetracycline (52), streptomycin (28), ampicillin (25), cephalothin (16), amoxicillin‐clavulanic acid (13), kanamycin (13), gentamycin (7), cefotaxime (3), ceftazidime (2), nalidixic acid (2), chloramphenicol (1), colistin (1), enrofloxacin (1)	Phenotypic	Italy	Razzuoli et al. (2021)
STEC	Chamois, red deer, roe deer	27	cephalosporins (27; *bla*_EC_)	Genotypic	Austria, Hungary, Poland, Slovenia, Switzerland	Nüesch‐Inderbinen et al. (2023)
	Chamois, fallow deer, red deer, roe deer	44	MDR (1): cefotaxime, ceftazidime, ciprofloxacin, colistin, gentamicin, meropenem, nalidixic acid, sulfamethoxazole	Phenotypic	Italy	Listorti et al. (2024)
	Red deer	4	None	Phenotypic	Spain	Díaz‐Sánchez et al. (2012)
	Wild boar	3	None	Phenotypic	Spain	Díaz‐Sánchez et al. (2012)
	Wild boar	3	cephalosporins (3; *bla*_EC_)	Genotypic	Switzerland	Nüesch‐Inderbinen et al. (2023)
*Listeria monocytogenes*	Wild boar	2	fosfomycin (2; NR^c^)	Genotypic	Italy	Floris et al. (2024)
*Yersinia enterocolitica*	Chamois	2	β‐lactams (2; NR), streptogramin (2; NR)	Genotypic	Italy	Floris et al. (2024)
	Red deer	1	β‐lactams (1; NR), streptogramin (1; NR)	Genotypic	Italy	Floris et al. (2024)
	Wild boar	5	β‐lactams (5; NR), streptogramin (5; NR)	Genotypic	Italy	Floris et al. (2024)
	Wild boar	126	ampicillin (108), sulfonamides (30), ceftiofur (9), streptomycin (1), tetracycline (1)	Phenotypic	Italy	Modesto et al. (2021)
	Wild boar	3	β‐lactams (3; *bla*A)	Phenotypic, Genotypic	Italy	Siddi et al. (2024)

*Note: N* = number of isolates.

Abbreviations: MDR, multi‐drug resistance; NR, not reported.

*Salmonella* isolates from wild boar meat exhibited highly variable AMR profiles, ranging from fully susceptible to highly resistant (Bonardi et al. 2021; Cilia et al. 2021; Razzuoli et al. 2021; Siddi et al. 2024). Resistance to streptomycin, sulfonamides, and tetracycline was frequently observed. One isolate (*S*. Infantis) was multi‐drug resistant (MDR) (Cilia et al. 2021).

AMR in STEC from game meat was investigated in three publications (Díaz‐Sánchez et al. 2012; Nüesch‐Inderbinen et al. 2023; Listorti et al. 2024), one of which employed whole‐genome sequencing to detect AMR genes (Nüesch‐Inderbinen et al. 2023). Based on phenotypic analyses, STEC isolates were generally susceptible to all tested antimicrobials (Table 5), except for one MDR isolate reported by Listorti et al. (2024) showing resistance to cefotaxime, ceftazidime, ciprofloxacin, colistin, gentamicin, meropenem, nalidixic acid, and sulfamethoxazole. Molecular analyses revealed the presence of the cephalosporinase‐encoding gene *bla*_EC_ in STEC isolates from Austria, Hungary, Poland, Slovenia, and Switzerland (Nüesch‐Inderbinen et al. 2023).

AMR was reported in two *L. monocytogenes* isolates, both of which were positive for genes coding for fosfomycin resistance (Floris et al. 2024). AMR in *Yersinia* isolates was studied in three publications (Modesto et al. 2021; Floris et al. 2024; Siddi et al. 2024). Most of the isolates were resistant to β‐lactams, including penicillins (ampicillin and amoxicillin) and first‐generation cephalosporins (Table 5). Frequent resistance to sulfamethoxazole‐trimethoprim was also reported (Modesto et al. 2021).

## 4 Discussion

### 4.1 Study Limitations and Identification of Data Gaps

This systematic review provides a comprehensive synthesis of the available evidence on the prevalence and virulence‐associated genes of bacterial zoonotic pathogens in hunted game meat while also identifying key data gaps in the current evidence base. Although the dataset was sufficiently large to support meaningful comparisons, notable gaps were identified for certain pathogens, particularly *Campylobacter*, for which relatively few studies were available.

The geographical distribution of available studies was highly uneven, with most publications originating from Europe and particularly Italy. An additional search including host species relevant to non‐European hunting systems identified only three additional eligible publications. Therefore, the limited geographical representation appears to reflect a genuine evidence gap rather than a major limitation of the search strategy. This finding itself is important, as it highlights the lack of information from large parts of Africa, Asia, the Americas, and Oceania.

Statistical modeling was constrained by the limited number of publications available for several pathogens and by the uneven distribution of studies across geographical regions, matrices, and sampling stages. Consequently, the analyses focused on the predefined factors of primary interest, namely detection method and animal group, while avoiding more highly parameterized models that could lead to unstable estimates.

Our search was restricted to studies published from 2004 onwards. Although this allowed the review to focus on evidence generated during the most recent two decades, relevant historical studies may have been excluded. Consequently, data extending beyond this period could not be evaluated.

Finally, detailed characterization of isolates, including WGS, virulence‐associated genes, serotyping, and AMR profiling was available for only a minority of studies. This limits the extent to which pathogen occurrence can be linked to public‐health relevance.

### 4.2 Methodological Considerations and Diagnostic Limitations

A key finding of this review is that methodological choices significantly influenced reported prevalence estimates. Molecular and culture‐based methods each have distinct advantages and limitations. In several publications, PCR was used as an initial screening tool due to its rapidity, cost‐effectiveness, and high analytical sensitivity, enabling the detection of bacterial DNA even in injured or viable non‐culturable cells. However, PCR‐based approaches may underestimate prevalence when inhibitors (e.g., hemoglobin and myoglobin, lipids, proteins) are present in complex matrices, such as meat, leading to false‐negative results (Fredriksson‐Ahomaa and Korkeala 2003). Conversely, reliance solely on culture‐based methods may also underestimate the true occurrence of pathogens, since stressed bacteria due to chilling, drying, or processing or viable but non‐culturable cells may escape detection (Kabwang a Mpalang et al. 2013; Sauvala et al. 2019). Importantly, PCR does not provide information on bacterial viability, and PCR‐positive findings should therefore not automatically be interpreted as evidence of viable pathogenic organisms. Studies that do not include culture confirmation may therefore overestimate the occurrence of pathogens. This limitation is particularly relevant when broad genetic markers are targeted, as these may also detect nonpathogenic strains. Consequently, methodological choices, including the selection of target genes and the use of confirmatory culture, strongly influence reported prevalence estimates and their interpretation in a public health context.

Across the studies, PCR‐based methods generally yielded higher detection rates than culture‐based methods. However, the magnitude and statistical significance of this difference varied among pathogens, which provides insight into pathogen‐specific diagnostic challenges. The larger discrepancies observed for STEC and *Yersinia* suggest that analytical choices, including molecular target selection and limitations of available isolation methods, may strongly influence the resulting prevalence estimates. In contrast, the smaller differences observed for *Listeria* indicate greater agreement between molecular and culture‐based approaches. While the sole use of culture‐based methods was most frequent for *Salmonella*, *Listeria*, and *Yersinia*, a combination of PCR and culture was more common for STEC and *Campylobacter*.

For *Y. enterocolitica*, the choice of the PCR target gene had a pronounced effect on the reported prevalence. As such, the highest *Y. enterocolitica* prevalence (75%) was observed using a PCR assay targeting the *yst*B gene (Peruzy, Cristiano, et al. 2022), which has been shown to be carried by nonpathogenic *Yersinia* spp. and non‐ or low‐pathogenic *Y. enterocolitica* 1A (Imori et al. 2017; Joutsen et al. 2020). By contrast, a substantially lower prevalence estimate (21%) was obtained when targeting *yst*A, which is associated with pathogenic *Y. enterocolitica*. These findings underscore the importance of selecting virulence‐associated markers, such as the *ail* and *ystA* genes, to ensure that PCR‐based detection reflects the presence of pathogenic *Y. enterocolitica*. Nevertheless, the high PCR‐based prevalence estimates reported using pathogenic markers should be interpreted as evidence of widespread detection of virulence‐associated genetic markers rather than direct evidence of viable pathogenic *Y. enterocolitica*. Nonetheless, PCR‐positive results for *Y. enterocolitica* were infrequently confirmed by culture (Fredriksson‐Ahomaa et al. 2020; Peruzy, Cristiano, et al. 2022; Peruzy, Murru, et al. 2022), which is partially attributable to the limited sensitivity of available culture methods for enteropathogenic *Yersinia*, which complicates the isolation and identification of pathogenic strains (Van Damme et al. 2013; Fredriksson‐Ahomaa 2023).

Particularly for STEC, the odds of detecting a positive sample were significantly higher with PCR than using culture. PCR screening of enrichment broths targeting the *stx* and *eae* genes often identified a substantial proportion of positive samples, though subsequent isolation rates were low (Nüesch‐Inderbinen et al. 2023). Consequently, PCR‐based detection primarily reflects the presence of STEC‐associated genetic markers, whereas public‐health assessment requires isolation of viable strains. Nevertheless, the isolation of STEC remains essential, as it enables detailed characterization of strains and assessment of their public health relevance. Although PCR methods may overestimate prevalence, the detection of STEC isolates is well known to be very difficult due to their similar morphology to (abundant) apathogenic *E. coli* on common media for *Enterobacteriaceae* (such as MacConkey agar and TBX agar) and even on chromogenic selective media (such as CHROMagar STEC).

For *L. monocytogenes*, detection predominantly relied on culture‐based methods, either alone or in combination with PCR. Although PCR‐based methods in the current study showed significantly higher odds of detecting *Listeria* than culture‐based methods, the effect size was modest and smaller than observed for *Salmonella*, STEC, or *Yersinia*. In studies employing both approaches, a high proportion of PCR‐positive samples were confirmed by culture, suggesting that culture methods for *L. monocytogenes* are comparatively sensitive (Sauvala et al. 2019; Fredriksson‐Ahomaa et al. 2020; Floris et al. 2024).

For *Salmonella*, PCR‐based methods also yielded higher detection estimates, although the effect was smaller than observed for STEC and *Yersinia*. This may indicate that conventional culture protocols, despite their widespread use, may underestimate *Salmonella* occurrence in complex matrices such as game meat, as high levels of background bacteria could hinder the enrichment and detection of *Salmonella* (Neyaz et al. 2024).

For *Campylobacter*, PCR and culture were used at similar frequencies. Although PCR‐based detection generally yielded higher prevalence estimates than culture‐based approaches, the difference was not statistically significant in the final models. This discrepancy between PCR and culture is consistent with the pathogen's known fastidious growth requirements (Park 2002). Moreover, background flora has also been shown to grow on classical *Campylobacter‐*selective media (Yoo et al. 2014) and can reduce the sensitivity of culture‐based methods (Abulreesh et al. 2005; Neyaz et al. 2024). High prevalence estimates in warthog and duiker meat based on combined PCR and culture methods indicate a substantial presence of viable *Campylobacter*, suggesting a genuinely elevated risk of human exposure rather than methodological overestimation within the specific studies and conditions analyzed.

To conclude, PCR offers a rapid and sensitive screening tool but may lead to overestimation or misinterpretation of public health risk if not carefully designed and interpreted, particularly when non‐virulence‐associated targets are used. Culture methods, while essential for confirming viability and enabling characterization, may underestimate prevalence due to methodological limitations. Therefore, an integrated approach combining PCR with targeted, virulence‐associated markers and subsequent culture confirmation is critical for generating accurate and meaningful prevalence data for properly assessing the associated public health risks. Moreover, beyond the distinction between PCR‐based and culture‐based methods, substantial heterogeneity existed in the analytical protocols themselves. Studies differed in enrichment procedures, selective media, PCR formats, primer and probe sets, and the extent of isolate characterization. Such methodological differences likely contributed to variability in prevalence estimates and complicate direct comparison between studies.

### 4.3 Pathogenic Potential and Characterization of Detected Isolates

A major limitation across the included studies is the insufficient characterization of isolates, which constrains the assessment of their public health relevance. In many cases, pathogen identification was not complemented by detailed analyses, such as virulence gene profiling, serotyping, or genomic characterization, limiting insights into their pathogenic potential and their relatedness to strains causing human disease. In addition, available data were confined to a small number of countries that performed such characterization.

Particularly for *Yersinia* spp., pathogenicity using chromosomal or plasmid‐associated virulence genes was not always assessed. Quite high isolation rates (12%–19%) of *Yersinia* spp. were found in the game meat of suids and ruminants, though most isolates had no or limited pathogenic potential based on the available virulence‐associated markers. The isolates predominantly belonged to *Y. enterocolitica* biotype 1A carrying the *yst*B gene, which is typically associated with nonpathogenic strains. By contrast, virulence determinants, such as *ail* and *yst*A, which are associated with pathogenic strains of *Y. enterocolitica*, were only sporadically detected in isolates from wild boar offal, moose and deer carcasses, and deer livers. Moreover, these *ail*‐positive isolates could not clearly be classified into any pathogenic biotype, and the presence of virulence plasmid‐encoded genes, such as *virF* and *yadA*, which is essential for full pathogenicity, were not reported. Nonpathogenic *ail*‐positive *Yersinia* isolates, including *Y. enterocolitica* and *Y. kristensenii*, have been found in wild animals (Joutsen et al. 2020). Genotypic analyses indicated that sequence types of *Y. enterocolitica* biotype 1A isolates from game meat differed from the types typically associated with human infections (Sauvala et al. 2019; Floris et al. 2024; Siddi et al. 2024). Isolates reported as biotype 1B were sporadically described in wild boar carcasses and livers from Poland and Italy. These isolates primarily carried *ystB* and lacked several virulence determinants. Because reliable distinction between biotypes 1A and 1B is challenging using phenotypic tests, and biotype determination was not supported by genomic characterization, the available data do not allow confirmation that classical highly pathogenic biotype 1B strains were present. Consequently, the pathogenic significance of these isolates should be interpreted with caution. Notably, *Y. enterocolitica* bioserotype 4/O3, which predominates domestic pigs, pork, and human yersiniosis cases, was not identified in any of the publications. These findings suggest that game meat is unlikely to represent a major source of pathogenic *Y. enterocolitica*. However, the detection of *Y. pseudotuberculosis* serotype O1 carrying the virulence plasmid‐associated *vir*F genes was found in wild boar offal (Fredriksson‐Ahomaa et al. 2020). These strains belonged to sequence type ST42, which has been associated with human infections in Europe.

For STEC, the pathogenic potential can be assessed through the identification of virulence‐associated genes, including *stx* and *eae* (World Health Organization 2019; EFSA Biohaz Panel et al. 2020; Lindqvist et al. 2023) as well as through genomic characterization (Blankenship et al. 2023). Particularly *stx*2a and *stx*2d are strongly associated with severe human disease, including HUS (Lindqvist et al. 2023; EFSA and ECDC 2025). Only two isolates detected in game meat from red deer in Spain (Díaz‐Sánchez et al. 2012) and wild boar in Switzerland (Nüesch‐Inderbinen et al. 2023) harbored high‐risk genomic profiles (i.e., *eae*‐/*stx*2a+). STEC isolates from game meat in the current study were generally characterized by virulence profiles that are less frequently associated with severe human disease. Most isolates harbored *stx2* with a clear predominance of the *stx*2*b* subtype, while *eae* was only rarely detected. The absence of *eae* suggests that these strains may rely on alternative adhesion factors for intestinal colonization, such as *lpfA* (long polar fimbriae A) (Torres et al. 2009) and *iha* (IrgA homologue adhesin) (Colello et al. 2019), which were carried by 70% and 78% of the STEC sequenced by Nüesch‐Inderbinen et al. (2023), respectively. The most common virulence gene profile observed in isolates from wild ungulate meat was *eae*‐/*stx*2b+, which is generally considered to have a lower virulence compared with profiles typically associated with severe human disease. This is further supported by serotyping. The major STEC serogroups most frequently implicated in human infections, commonly referred to as the “Top 7” (O26, O45, O103, O111, O121, O145, and O157) (EFSA and ECDC 2025), were rarely detected in game meat

Most STEC isolates from game meat harbored virulence gene profiles that are less frequently associated with severe human disease. Nevertheless, the presence of specific serogroups, sequence types, and virulence determinants reported among human clinical isolates were identified, demonstrating that game meat can contain strains with recognized pathogenic potential.

The isolation rate of *Listeria* spp. was high in wild boar carcasses in one study (Stella et al. 2018); however, no *L. monocytogenes* was identified, highlighting the importance of species‐level identification for assessing the public health risk. For *L. monocytogenes*, serogroup IIa was the most frequently identified serogroup in this review. Serogroup IIa is a frequent type among both human and food isolates, but the proportion is significantly higher among food isolates (Suominen et al. 2023). By contrast, serogroup IVb, which is more often associated with severe human listeriosis and outbreaks, was only rarely detected in game meat. Game meat has been implicated in *L. monocytogenes* outbreaks using MLST (Filipello et al. 2020). Available data on sequence types were very limited in this review, but several game meat sequence types have been associated with human listeriosis (Fredriksson‐Ahomaa et al. 2022), indicating that isolates from game meat may pose a public health risk. However, the limited availability of whole genome sequence (WGS) data constrains comprehensive conclusions.

*S. enterica* subspecies *enterica* was the most frequently identified subspecies in wild boar meat, though the other five subspecies were also reported. A wide variety of *Salmonella* serotypes were reported, with *S*. Typhimurium, *S*. Enteritidis, *S*. Coeln, *S*. Thompson and *S*. Newport being among the most frequent. Importantly, several of these serotype are also among the leading causes of human salmonellosis (EFSA and ECDC 2024), indicating that game meat may harbor strains with established relevance for human disease. However, data on isolate characterization in the current study was limited. Similarly, several outbreaks that have been associated with game meat lacked molecular confirmation connecting human and game meat derived isolates (Kuhn et al. 2011; Madar et al. 2012; Draper et al. 2025). Overall, the available characterization data indicate that game meat may harbor pathogens with demonstrated public health relevance, but the lack of comprehensive genomic characterization and source attribution studies limits definitive conclusions regarding transmission to humans.

Species‐level identification of *Campylobacter* isolates indicates that *C. coli* was the dominant species in game meat, including smoked products, across both suids and ruminants. However, *C. jejuni*, a major human pathogen, was also consistently detected, albeit less frequently, highlighting its relevance for food safety (Kabwang a Mpalang et al. 2013). Its occurrence, particularly in insufficiently heat‐treated products, such as smoked meat, underscores a potential risk to consumers (Kabwang a Mpalang et al. 2013). In addition, the detection of *C. lanienae* points to the broader diversity of *Campylobacter* species in game meat. Although its public health significance is less well understood, its presence alongside *C. coli* and *C. jejuni* in wild boar offal, including the liver, suggests that game meat may serve as a reservoir for multiple *Campylobacter* species.

Across all investigated pathogens, a consistent limitation is the insufficient characterization of isolates with respect to virulence determinants, serogroups, and genomic profiles. While many isolates detected in game meat appear to have a lower pathogenic potential compared with those commonly associated with human disease, the presence of specific virulence factors, serogroups, and sequence types linked to human infections indicate that game meat cannot be excluded as a potential source of transmission. Clear epidemiological links between game meat consumption and human illness are rarely established, and available genomic data remain limited. This highlights the need for more comprehensive surveillance, including WGS and virulence gene profiling.

### 4.4 Matrix and Sampling Stage

Comparing prevalence estimates across studies was not only constrained by analytical methods, but also by substantial heterogeneity in the sample matrices and sampling stages. The type of matrix analyzed (e.g., carcass swabs, muscle tissue, offal, or processed products) and the point of sampling along the production chain (hunting site, processing, or retail) represent different combinations of primary contamination, cross‐contamination, and potential bacterial growth during storage. Consequently, the prevalence estimates reported in this review should not be interpreted as representing a single stage of the game‐meat chain. Instead, they reflect occurrence in meat sampled at different points, ranging from carcasses immediately after hunting to retail products. The pooled prevalence estimates should therefore be interpreted as indicators of pathogen occurrence rather than direct measure of consumer exposure.

Several studies included in this review suggested that carcass contamination may be influenced by factors related to hunting and field dressing practices, including shooting accuracy, time to evisceration, carcass handling, and hygiene during transport and processing (Atanassova et al. 2008; Avagnina et al. 2012; Mirceta et al. 2017; Stella et al. 2018; Sauvala et al. 2019; Branciari et al. 2020; Cenci‐Goga et al. 2020; Nkosi et al. 2022; Peruzy, Murru, et al. 2022). Likewise, studies sampling meat at processing or retail stages reflect not only field contamination but also the effects of subsequent handling, processing, packaging, storage, and opportunities for cross‐contamination (Ranucci et al. 2021; Bonardi et al. 2021; Sauvala et al. 2023; Kabwang a Mpalang et al. 2013).

The variation observed between studies is therefore likely to reflect a combination of biological differences, hunting practices, processing conditions and analytical methodology. However, because most studies investigated only a single matrix at a single stage of the production chain, the independent effects of matrix type and sampling stage could not be quantified reliably. This limitation likely contributed substantially to the heterogeneity observed across prevalence estimates. Despite these limitations, the available evidence consistently supports the importance of hygienic field dressing practices, minimization of fecal contamination during carcass handling, and adequate hygiene and temperature control during processing and storage to reduce contamination risks throughout the game‐meat chain.

### 4.5 Influence of Animal Groups

Across pathogens, a consistent pattern emerged in which prevalence estimates were generally higher in meat from suids than from wild ruminants, with the notable exception of STEC. These differences likely reflect differences in ecology, behavior, and gastrointestinal physiology between the two host groups.

Wild boars exhibit omnivorous and opportunistic feeding behavior, including rooting and scavenging, which increases exposure to a wide range of environmental and fecal contaminants (Carlos et al. 2023), facilitating pathogen acquisition and transmission of enteric pathogens. In line with this, our results indicate that wild boar meat represents a more important source of *Salmonella* compared with ruminant game meat, for which the prevalence was consistently low. Nevertheless, considerable variation exists, and low prevalence in suis has also been reported (Mirceta et al. 2017; Petrovic et al. 2024), suggesting that beyond host ecology, factors such as hunting hygiene also play an important role. The importance of suids in the epidemiology of *Salmonella* and *Yersinia* is further supported by their recognized role as reservoirs in domestic pigs.

By contrast, STEC showed a different pattern, with a higher prevalence in ruminant game meat compared with suids. This is consistent with the well‐established role of domestic ruminants as major reservoirs of STEC (Mughini‐Gras et al. 2018; Pires et al. 2019). Host‐specific adaptations facilitating long‐term intestinal colonization likely explain why STEC is more strongly associated with ruminant game than with meat from suids (Kudva and Stasko 2013; Matthews et al. 2006; Segura et al. 2021).

In this systematic review, *L. monocytogenes* was reported in the game meat of both suids and ruminants, showing that game meat can be a source of *L. monocytogenes*. While most studies reported relatively low prevalence estimates (< 8%), occasional high prevalence rates were reported in both ruminant and suid game meat. *L. monocytogenes* is ubiquitous in soil, which may reflect the higher prevalence in suids. However, given the importance of post‐harvest contamination and bacterial growth for this pathogen, differences between host species should be interpreted with caution. Regarding *Yersinia*, the prevalence was significantly higher in suid meat compared with ruminants. Wild suids commonly eat rodents and birds, which frequently carry and transmit *Yersinia* in their intestinal tract. In African settings, the predominance of *C. coli* may be linked to its association with suids and wildlife, combined with informal meat production practices, such as bushmeat processing, limited hygiene, and insufficient heat treatment, which facilitate contamination and bacterial survival. This is further supported by clinical data from Ghana (Karikari et al. 2017), where *C. coli* accounted for 37% of all isolates and was the predominant species in patients with gastroenteritis (46%), highlighting its significant role in human infections in the region.

### 4.6 Antimicrobial Resistance

Although AMR was not the primary focus of this review and available data were limited, the detection of antimicrobial‐resistant foodborne pathogens in game meat remains relevant because these bacteria may be transmitted to consumers or food handlers. AMR data were reported in a limited number of studies, so interpretation of these findings requires caution. Bacteria isolated from game meat may reflect contamination originating from the animal itself, environmental sources, or processing‐related cross‐contamination. Consequently, the available data in this review do not allow robust conclusions regarding the role of wildlife as reservoirs of AMR.

For *Salmonella*, reported resistance profiles in wild boar meat ranged from fully susceptible to resistant isolates, with variability likely reflecting both regional differences and limited sample sizes. In general, AMR in *Salmonella* is known to depend on geographical location, source, and serotype (Wang et al. 2025). Resistance to older antimicrobials, such as streptomycin, sulfonamides, and tetracycline, was relatively common, whereas resistance to critically important antimicrobials for human medicine, including fluoroquinolones and third‐generation cephalosporins, remained rare (Cilia et al. 2021; Razzuoli et al. 2021). Nevertheless, evidence from wild boar feces suggests that higher AMR levels, including resistance to critically important antimicrobials, may occur (Akwongo et al. 2025), highlighting the potential for carcass contamination under inadequate hygiene conditions.

For STEC, AMR data were particularly scarce and indicated a predominantly susceptible phenotype. Only a single MDR isolate was reported (Listorti et al. 2024), while other isolates were fully susceptible. The detection of chromosomal *bla*_EC_ genes in STEC from game meat (Nüesch‐Inderbinen et al. 2023) suggests the potential for resistance to third‐generation cephalosporins; however, their phenotypic expression was not assessed. Overall, these findings contrast with higher resistance levels reported in STEC and commensal *E. coli* from conventional meat sources (Rega et al. 2021; EFSA and ECDC 2026), indicating that game meat may currently represent a less important reservoir of AMR in this pathogen.

Very limited information was available for *L. monocytogenes*, with AMR assessed in only two isolates (Floris et al. 2024). Genes coding for fosfomycin resistance were detected in both isolates. Fosfomycin resistance is considered intrinsic to the species and is therefore of limited clinical relevance (Fredriksson‐Ahomaa et al. 2022; Díaz‐Martínez et al. 2025). One isolate showed resistance to cefotaxime and nalidixic acid, two clinically critical antimicrobials. Similarly, for *Y. enterocolitica*, much of the observed resistance, particularly to β‐lactams, can be attributed to intrinsic mechanisms, including chromosomal *bla* genes (Koskinen et al. 2022; Siddi et al. 2024) and the chromosomal *vat*F gene for streptogramin resistance (Floris et al. 2024). When intrinsic resistance was excluded, acquired resistance and MDR appeared to be uncommon, although sporadic MDR isolates have been reported (Modesto et al. 2021).

### 4.7 Public Health Relevance

The occurrence of foodborne pathogens in game meat should not be interpreted directly as consumer risk. Public health risk depends not only on pathogen prevalence, but also on bacterial viability, concentration in food, pathogenic characteristics of the strains present, preparation practices, consumption patterns, and the susceptibility of the exposed population. Consequently, the prevalence estimates reported in this review should be regarded as indicators of potential exposure rather than direct measures of public health risk.

The public health relevance differed between pathogens. For *Salmonella*, several serotypes identified in game meat overlapped with those frequently associated with human salmonellosis, suggesting that game meat may act as a vehicle for strains relevant to human health. In contrast, the available evidence for *Yersinia* indicated that most isolates belonged to biotype 1A or lacked key virulence‐associated markers, whereas the biotypes most commonly associated with human yersiniosis were rarely or never detected. These findings suggest that game meat is unlikely to represent a major source of classical pathogenic *Y. enterocolitica*, although sporadic detection of pathogenic *Yersinia* species demonstrates that transmission remains possible.

For STEC, most characterized isolates harbored virulence profiles that are less frequently associated with severe human disease than those commonly reported from cattle. Nevertheless, isolates carrying virulence‐associated genes, serogroups, and sequence types linked to human disease were identified, indicating that game meat cannot be excluded as a potential source of clinically relevant STEC. Likewise, *L. monocytogenes* isolates primarily belonged to serogroups commonly found in food, while highly pathogenic outbreak‐associated serogroups were reported only sporadically. However, given the ability of *L. monocytogenes* to survive and grow during refrigerated storage, even relatively low contamination levels may be relevant under unfavorable storage conditions.

The public health significance of *Campylobacter* remains difficult to assess because only a limited number of studies were available. The detection of both *C. jejuni* and *C. coli* demonstrates that recognized human pathogens can occur in game meat, but insufficient data currently exist to demonstrate the relative importance of game meat as a transmission route.

AMR was only sporadically reported and multidrug‐resistant isolates were uncommon. Nevertheless, the detection of multidrug‐resistant *Salmonella*, STEC, and *Yersinia* isolates demonstrates that game meat may contribute to environmental pathways for the dissemination of AMR and highlights the value of continued surveillance.

## 5 Conclusions

This systematic review demonstrates that hunted game meat can be contaminated with zoonotic bacterial pathogens capable of causing disease in consumers. However, the magnitude of this contribution of game meat is difficult to assess due to the limited number of available studies, particularly for *Campylobacter*. The substantial heterogeneity among included studies investigating *Salmonella*, STEC, *L. monocytogenes, Yersinia*, and *Campylobacter*, especially regarding the analytical methodologies, animal species, and sample matrices tested, the limited information on pathogenicity and characterization of most pathogens, and the various geographical areas, highlights the need for more harmonized data to enable more robust and accurate risk assessments. We therefore recommend increasing the number of studies on hunted game meat and placing greater emphasis on the characterization of isolates, particularly with respect to pathogenic potential and AMR, to better evaluate the public health significance of pathogens detected in game meat. Moreover, additional research is needed in countries and regions where game meat is commonly consumed to achieve a more comprehensive and globally representative understanding of the microbiological safety of wild game meat.

## Author Contributions

**Maria Fredriksson‐Ahomaa**: conceptualization, methodology, investigation, writing – review and editing, writing – original draft, visualization, supervision, project administration. **Silvia Bonardi**: conceptualization, investigation, writing – original draft, writing – review and editing. **Bozena Futoma‐Koloch**: investigation, writing – original draft, writing – review and editing. **Ioannis Sakaridis**: investigation, writing – original draft, writing – review and editing. **Jelena Petrovic**: investigation, writing – original draft, writing – review and editing. **Inge Van Damme**: data curation, methodology, investigation, validation, formal analysis, visualization, writing – original draft, writing – review and editing.

## Funding

Proofreading and open access funding were provided by the University of Helsinki.

## Conflicts of Interest

The authors declare no conflicts of interest.

## Supporting information




**Supplementary Material**: crf370657‐sup‐0001‐AppendixS1‐S11.pdf

## Data Availability

The dataset will be deposited in an open access repository, such as Zenodo (https://doi.org/10.5281/zenodo.20054849). Note to the journal/reviewers: The database can already be previewed via this link. The database will be published after acceptance of this paper.
