# Supplementary material for: Pathogenic Foodborne Bacteria in the Game Meat of Hunted Large Ungulates—A Systematic Review and Meta‐Analysis
Source: Compr Rev Food Sci Food Saf. 2026 Sep 18;25(5):e70657. doi: 10.1111/1541-4337.70657 (PMC13589185; doi:10.1111/1541-4337.70657)
Supplement: Supplementary file 1 [file CRF3-25-e70657-s001.pdf]

Appendix - Pathogenic foodborne bacteria in the game meat of hunted large ungulates — a systematic review and meta-analysis

Contents

Table S1 | Database-specific search strings. 2

Table S2 | Bias assessment results using the Joanna Briggs Institute Critical Appraisal checklist. 3

Table S3 | Characteristics of publications included in the review (n=46). 4

Table S4 | Number of publications reporting the prevalence of foodborne pathogenic bacteria in meat of hunted ungulates according to the animal group. 6

Table S5 | Number of publications reporting the prevalence of foodborne pathogenic bacteria in meat of hunted ungulates according to the sample matrix. 6

Table S6 | Number of publications reporting the prevalence of foodborne pathogenic bacteria in meat of hunted ungulates according to the detection method that was used. 6

Table S7 | Overview of studies reporting the prevalence of *Salmonella* spp. in game meat of wild hunted ungulates. 7

Table S8 | Overview of studies reporting the prevalence of STEC in game meat of wild hunted ungulates. 9

Table S9 | Overview of studies reporting the prevalence of *Listeria* spp. in game meat of wild hunted ungulates. 10

Table S10 | Overview of studies reporting the prevalence of *Yersinia* spp. in game meat of wild hunted ungulates. 11

Table S11 | Overview of studies reporting the prevalence of Campylobacter spp. in game meat of wild hunted ungulates. 12

Table S1 | Database-specific search strings.

Database	Search string
PubMed	*Initial search (January 2025):*<br>(((deer) OR (ibex) OR (chamois) OR (moose) OR (elk) OR (wild AND boar) OR (ungulate)) AND (meat OR carcass OR carcase OR organ OR liver OR offal) AND (salmonella OR campylobacter OR yersinia OR stec OR vtec OR escherichia)) AND (("2004/01/01"[Date - Publication] : "3000"[Date - Publication]))<br>Translation: ("deer"[MeSH Terms] OR "deer"[All Fields] OR "ibex"[All Fields] OR ("rupicapra"[MeSH Terms] OR "rupicapra"[All Fields] OR "chamois"[All Fields]) OR ("moose"[All Fields] OR "mooses"[All Fields]) OR "elk"[All Fields] OR ("wild"[All Fields] AND ("swine"[MeSH Terms] OR "swine"[All Fields] OR "boar"[All Fields])) OR ("ungulate"[All Fields] OR "ungulates"[All Fields])) AND ("meat"[MeSH Terms] OR "meat"[All Fields] OR ("carcass"[All Fields] OR "carcasses"[All Fields]) OR ("carcase"[All Fields] OR "carcases"[All Fields]) OR ("organ"[All Fields] OR "organ s"[All Fields] OR "organism"[All Fields] OR "organism s"[All Fields] OR "organisms"[All Fields] OR "organs"[All Fields]) OR ("liver"[MeSH Terms] OR "liver"[All Fields] OR "livers"[All Fields] OR "liver s"[All Fields]) OR ("offal"[All Fields] OR "offals"[All Fields])) AND ("salmonella"[MeSH Terms] OR "salmonella"[All Fields] OR "salmonellas"[All Fields] OR "salmonella s"[All Fields] OR "salmonellae"[All Fields] OR ("campylobacter"[MeSH Terms] OR "campylobacter"[All Fields] OR "campylobacters"[All Fields]) OR ("yersinia"[MeSH Terms] OR "yersinia"[All Fields] OR "yersinia infections"[MeSH Terms] OR ("yersinia"[All Fields] AND "infections"[All Fields]) OR "yersinia infections"[All Fields] OR "yersiniae"[All Fields] OR "yersinias"[All Fields]) OR ("shiga toxigenic escherichia coli"[MeSH Terms] OR ("shiga toxigenic"[All Fields] AND "escherichia"[All Fields] AND "coli"[All Fields]) OR "shiga toxigenic escherichia coli"[All Fields] OR "stec"[All Fields]) OR ("shiga toxigenic escherichia coli"[MeSH Terms] OR ("shiga toxigenic"[All Fields] AND "escherichia"[All Fields] AND "coli"[All Fields]) OR "shiga toxigenic escherichia coli"[All Fields] OR "vtec"[All Fields]) OR ("escherichia"[MeSH Terms] OR "escherichia"[All Fields] OR "escherichiae"[All Fields])) AND 2004/01/01:3000/12/31[Date - Publication]<br>*Additional search (March 2026):*<br>(antelope OR muntjac OR impala OR springbok OR wildebeest OR duiker OR reindeer OR buffalo OR bison OR ibex OR caribou OR (feral AND (hog OR pig)) OR bushmeat) AND (meat OR carcass OR carcase OR organ OR offal OR liver) AND (salmonella OR campylobacter OR yersinia OR stec OR listeria) Filters: 2004 - 2025
Scopus	*Initial search (January 2025):*<br>(TITLE-ABS-KEY (((deer) OR (ibex) OR (chamois) OR (moose) OR (elk) OR (wild AND boar) OR (ungulate)) AND (meat OR carcass OR carcase OR organ OR liver OR offal) AND (salmonella OR campylobacter OR yersinia OR stec OR vtec OR escherichia))) AND (LIMIT-TO (SRCTYPE , "j")) AND (LIMIT-TO (DOCTYPE , "ar")) AND (LIMIT-TO (LANGUAGE , "English"))<br>*Additional search (March 2026):*<br>(antelope OR muntjac OR impala OR springbok OR wildebeest OR duiker OR reindeer OR buffalo OR bison OR ibex OR caribou OR (feral AND (hog OR pig)) OR bushmeat) AND (meat OR carcass OR carcase OR organ OR offal OR liver) AND (salmonella OR campylobacter OR yersinia OR stec OR listeria) Filters: 2004-2025
Google Scholar	Initial search (January 2025):<br>(((deer) OR (ibex) OR (chamois) OR (moose) OR (elk) OR (wild AND boar) OR (ungulate)) AND (meat OR carcass OR carcase OR organ OR liver OR offal) AND (salmonella OR campylobacter OR yersinia OR stec OR vtec OR escherichia)) Filters: from 2004-2025<br>*Additional search (March 2026):*<br>(antelope OR muntjac OR impala OR springbok OR wildebeest OR duiker OR reindeer OR buffalo OR bison OR ibex OR caribou OR (feral AND (hog OR pig)) OR bushmeat) AND (meat OR carcass OR carcase OR organ OR offal OR liver) AND (salmonella OR campylobacter OR yersinia OR stec OR listeria) Filters: 2004-2025

Table S2 | Bias assessment results using the Joanna Briggs Institute Critical Appraisal checklist.
Bias score = overall bias score; Q1 = Was the sample frame appropriate to address the target population?; Q2 = Were study participants sampled in an appropriate way?; Q3 = Was the sample size adequate?; Q4 = Were the study subjects and the setting described in detail?; Q5 = Was the data analysis conducted with sufficient coverage of the identified sample?; Q6 = Were valid methods used for the identification of the condition?; Q7 = Was the condition measured in a standard, reliable way for all participants?; Q8 = Was there appropriate statistical analysis?; Q9 = Was the response rate adequate, and if not, was the low response rate managed appropriately? (not considered applicable for this review)

Reference	Bias score	Q1	Q2	Q3	Q4	Q5	Q6	Q7	Q8
Eglezos et al. (2008)	8	Yes	Yes	Yes	Yes	Yes	Yes	Yes	Yes
Conedera et al. (2014)	5	Yes	Unclear	Yes	Yes	Yes	Yes	Yes	No
Citterio et al. (2014)	7	Yes	Yes	Yes	No	Yes	Yes	Yes	Yes
Weindl et al. (2016)	6	Yes	Yes	No	Yes	Yes	Yes	Yes	No
Giorda et al. (2014)	5	Yes	Yes	Unclear	No	Yes	No	Yes	Yes
Rîmbu et al. (2015)	3	Yes	Unclear	Unclear	No	Unclear	Yes	No	Yes
Ludwiczak et al. (2019)	6	Yes	Yes	No	Yes	Yes	Yes	Yes	No
Kabwang a Mpalang et al. (2013)	8	Yes	Yes	Yes	Yes	Yes	Yes	Yes	Yes
Elezi et al. (2013)	5	Yes	Unclear	No	No	Yes	Yes	Yes	Yes
Mignone et al. (2015)	4	Yes	Unclear	Yes	No	Unclear	Yes	Yes	No
Sauvala et al. (2023)	6	Yes	Yes	No	No	Yes	Yes	Yes	Yes
Siddi et al. (2023)	8	Yes	Yes	Yes	Yes	Yes	Yes	Yes	Yes
Sauvala et al. (2019)	8	Yes	Yes	Yes	Yes	Yes	Yes	Yes	Yes
Ziomek et al. (2023)	7	Yes	Yes	Yes	Yes	Unclear	Yes	Yes	Yes
Fredriksson-Ahomaa et al. (2020)	8	Yes	Yes	Yes	Yes	Yes	Yes	Yes	Yes
Orsoni et al. (2020)	8	Yes	Yes	Yes	Yes	Yes	Yes	Yes	Yes
Peruzy, Murru, et al. (2022)	8	Yes	Yes	Yes	Yes	Yes	Yes	Yes	Yes
Modesto et al. (2021)	7	Yes	Yes	Yes	No	Yes	Yes	Yes	Yes
Peruzy, Cristiano, et al. (2022)	7	Yes	Yes	Yes	Yes	Unclear	Yes	Yes	Yes
Thierry et al. (2020)	8	Yes	Yes	Yes	Yes	Yes	Yes	Yes	Yes
Altissimi et al. (2024)	8	Yes	Yes	Yes	Yes	Yes	Yes	Yes	Yes
Hingston et al. (2020)	5	Yes	Unclear	No	No	Yes	Yes	Yes	Yes
Ranucci et al. (2021)	8	Yes	Yes	Yes	Yes	Yes	Yes	Yes	Yes
Petrović et al. (2024)	7	Yes	Yes	Yes	Yes	Unclear	Yes	Yes	Yes
Bonardi et al. (2021)	8	Yes	Yes	Yes	Yes	Yes	Yes	Yes	Yes
Asakura et al. (2017)	8	Yes	Yes	Yes	Yes	Yes	Yes	Yes	Yes
Cilia et al. (2021)	8	Yes	Yes	Yes	Yes	Yes	Yes	Yes	Yes
Sannö et al. (2023)	8	Yes	Yes	Yes	Yes	Yes	Yes	Yes	Yes
Listorti et al. (2024)	8	Yes	Yes	Yes	Yes	Yes	Yes	Yes	Yes
Floris et al. (2024)	7	Yes	Yes	Yes	Yes	Yes	Yes	Yes	Unclear
Stella et al. (2018)	8	Yes	Yes	Yes	Yes	Yes	Yes	Yes	Yes
Nüesch-Inderbinen et al. (2023)	8	Yes	Yes	Yes	Yes	Yes	Yes	Yes	Yes
Branciari et al. (2020)	8	Yes	Yes	Yes	Yes	Yes	Yes	Yes	Yes
Razzuoli et al. (2021)	4	Yes	Unclear	Yes	No	Yes	Unclear	Unclear	Yes
Peruzy et al. (2019)	7	Yes	Yes	No	Yes	Yes	Yes	Yes	Yes
Magwedere et al. (2013)	5	Yes	Unclear	No	No	Yes	Yes	Yes	Yes
Paulsen and Winkelmayer (2004)	6	Yes	Yes	Yes	No	Unclear	Yes	Yes	Yes
Mirceta et al. (2017)	7	Yes	Yes	Yes	Yes	Unclear	Yes	Yes	Yes
Avagnina et al. (2012)	8	Yes	Yes	Yes	Yes	Yes	Yes	Yes	Yes
Bancerz-Kisiel et al. (2016)	7	Yes	Yes	Yes	Yes	Yes	Yes	Yes	No
Atanassova et al. (2008)	7	Yes	Yes	Yes	Yes	Yes	Yes	Yes	No
Díaz-Sánchez et al. (2013)	8	Yes	Yes	Yes	Yes	Yes	Yes	Yes	Yes
Díaz-Sánchez et al. (2012)	6	Yes	Yes	Yes	No	Unclear	Yes	Yes	Yes
Bachand et al. (2012)	6	Yes	No	Yes	Yes	Yes	Yes	No	Yes
Haindongo et al. (2018)	7	Yes	Yes	Yes	No	Yes	Yes	Yes	Yes
Nkosi et al. (2022)	6	Yes	Yes	Yes	Yes	Yes	Yes	No	No

Table S3 | Characteristics of publications included in the review (n=46).

Reference	N[a]	Country	Sampling year(s)	Pathogen(s)	Animal species
Altissimi et al. (2024)	2	Italy	2018-2023	*Salmonella*	Wild boar
Asakura et al. (2017)	6	Japan	2015-2016	STEC, *Salmonella*	Deer (not specified), Wild boar
Atanassova et al. (2008)	9	Germany	2006-2007	*Salmonella*, *Campylobacter*, *Listeria*	Wild boar, Western roe deer, Red deer
Avagnina et al. (2012)	9	Italy	2008-2010	*Salmonella*, *Yersinia*, *Listeria*	Wild boar, Chamois, Western roe deer, Red deer, Multiple species
Bachand et al. (2012)	4	Gabon	2010	*Salmonella*, *Campylobacter*	Duikers, Red river hog
Bancerz-Kisiel et al. (2016)	3	Poland	2013	*Yersinia*	Western roe deer, Red deer, Wild boar
Bonardi et al. (2021)	2	Italy	2020	*Salmonella*, *Yersinia*	Wild boar
Branciari et al. (2020)	2	Italy	2018-2019	*Salmonella*, *Listeria*	Western roe deer
Cilia et al. (2021)	1	Italy	2018-2020	*Salmonella*	Wild boar
Citterio et al. (2014)	10	Italy	2010-2012	*Salmonella*, *Listeria*	Chamois, Western roe deer, Red deer, Mouflon, Wild boar
Conedera et al. (2014)	1	Italy	2012-2013	*Salmonella*	Wild boar
Díaz-Sánchez et al. (2012)	4	Spain	2010-2011	STEC	Wild boar, Red deer
Díaz-Sánchez et al. (2013)	6	Spain	2009-2011	*Salmonella*, STEC	Wild boar, Red deer
Eglezos et al. (2008)	1	Australia	2004-2006	*Salmonella*	Wild boar
Elezi et al. (2013)	1	Albania	not specified	*Salmonella*	Wild boar
Floris et al. (2024)	32	Italy	2020-2022	*Salmonella*, *Listeria*, *Yersinia*, STEC	Chamois, Western roe deer, Red deer, Wild boar
Fredriksson-Ahomaa et al. (2020)	9	Finland	2016	*Campylobacter*, *Salmonella*, *Yersinia*, *Listeria*, STEC	Wild boar
Giorda et al. (2014)	3	Italy	2011-2013	*Salmonella*	Wild boar, Chamois, Western roe deer
Haindongo et al. (2018)	4	Namibia	2016	STEC	Springbok, Gemsbok, Greater kudu, Wildebeest
Hingston et al. (2020)	4	Canada	not specified	*Salmonella*, *Listeria*	Eurasian elk, Deer (not specified)
Kabwang a Mpalang et al. (2013)	18	DRC	2009-2010	STEC, *Salmonella*, *Campylobacter*	African buffalo, Warthog, Duikers
Listorti et al. (2024)	2	Italy	2019-2023	STEC	Multiple species (Roe deer, Fallow deer, Red deer, Chamois)
Ludwiczak et al. (2019)	2	Poland	2017	*Salmonella*, *Listeria*	Wild boar
Magwedere et al. (2013)	5	US	not specified	STEC	White-tailed deer, Red deer, Reindeer, American bison, Wild boar
Mignone et al. (2015)	2	Italy	2013-2015	*Yersinia*, *Campylobacter*	Wild boar
Mirceta et al. (2017)	1	Serbia	2015	*Salmonella*	Wild boar
Modesto et al. (2021)	1	Italy	2013-2018	*Yersinia*	Wild boar

Reference	N[a]	Country	Sampling year(s)	Pathogen(s)	Animal species
Nkosi et al. (2022)	1	South Africa	not specified	*Salmonella*	Impala
Nüesch-Inderbinen et al. (2023)	8	Multiple[b]	2021	STEC	Chamois, Red deer, Western roe deer, Wild boar
Orsoni et al. (2020)	1	Italy	2015	*Salmonella*	Wild boar
Paulsen and Winkelmayer (2004)	10	Austria	2003	*Salmonella*, *Listeria*	Red deer, Western roe deer, Chamois, Wild boar, Mouflon
Peruzy et al. (2019)	5	Italy	2017	*Salmonella*, *Listeria*, *Yersinia*	Wild boar
Peruzy, Cristiano, et al. (2022)	8	Italy	2019-2020	*Salmonella*, *Yersinia*, STEC, *Campylobacter*	Wild boar
Peruzy, Murru, et al. (2022)	8	Italy	2019	*Salmonella*, *Yersinia*, *Campylobacter*, STEC	Wild boar
Petrović et al. (2024)	1	Serbia	2013-2014	*Salmonella*	Wild boar
Ranucci et al. (2021)	1	Italy	2018-2019	*Salmonella*	Wild boar
Razzuoli et al. (2021)	2	Italy	2013-2017	*Salmonella*	Wild boar
Rîmbu et al. (2015)	2	Romania	not specified	*Salmonella*, *Listeria*	Wild boar
Sannö et al. (2023)	1	Sweden	not specified	*Yersinia*	Wild boar
Sauvala et al. (2019)	16	Finland	2012-2015	*Campylobacter*, *Salmonella*, STEC, *Yersinia*, *Listeria*	Eurasian elk, White-tailed deer
Sauvala et al. (2023)	5	Finland	2018	*Campylobacter*, *Salmonella*, STEC, *Yersinia*, *Listeria*	White-tailed deer
Siddi et al. (2023)	2	Italy	2020-2022	*Salmonella*, *Yersinia*	Wild boar
Stella et al. (2018)	3	Italy	not specified	*Listeria*, *Campylobacter*, *Salmonella*	Wild boar
Thierry et al. (2020)	1	Mauritius	2015-2017	STEC	Rusa deer
Weindl et al. (2016)	2	Germany	2011-2012	*Listeria*	Wild boar, Red deer
Ziomek et al. (2023)	2	Italy	2018-2019	*Campylobacter*	Wild boar

[a]N: number of prevalence estimates extracted from the publication; [b]Multiple countries: Austria, Croatia, Germany, Hungary, Poland, Slovenia, Switzerland.

Table S4 | Number of publications reporting the prevalence of foodborne pathogenic bacteria in meat of hunted ungulates according to the animal group.

Pathogen	Number of publications	Suids only (N=23)	Ruminants only (N=8)	Both ruminants and suids (N=15)
Salmonella	34	19	5	10
STEC	15	3	5	7
Listeria	15	5	4	6
Yersinia	14	9	2	3
Campylobacter	11	6	2	3

Table S5 | Number of publications reporting the prevalence of foodborne pathogenic bacteria in meat of hunted ungulates according to the sample matrix.

Pathogen	Number of publications	Carcass meat (N=22)	Cut meat and meat products (N=15)	Edible offal (N=12)
Salmonella	34	19	10	7
STEC	15	4	8	3
Listeria	15	8	4	3
Yersinia	14	6	4	4
Campylobacter	11	6	3	3

Table S6 | Number of publications reporting the prevalence of foodborne pathogenic bacteria in meat of hunted ungulates according to the detection method that was used.

Pathogen	Number of publications	Culture-based isolation (N=27)	PCR screening+ isolation (N=13)	PCR detection (N=4)	ELFA[a] screening + isolation (N=2)
Salmonella	34	23	8	1	2
STEC	15	1	9	5	0
Listeria	15	9	4	1	1
Yersinia	14	7	4	3	0
Campylobacter	11	5	5	1	0

[a]ELFA: enzyme-linked fluorescent immunoassay (VIDAS); for STEC, one publication using ELFA screening was excluded because the risk of bias was too high since the assay only targeted serotype O157:H7, which is rarely reported in wild game meat.

Table S7 | Overview of studies reporting the prevalence of *Salmonella* spp. in game meat of wild hunted ungulates.

Reference	Animal group	Number of samples	Culture results	PCR results	Animal species	Matrix
Altissimi et al. (2024)	Suids	226	5 (2%)	NA	Wild boar	Carcass
Altissimi et al. (2024)	Suids	258	3 (1%)	NA	Wild boar	Edible offal
Asakura et al. (2017)	Suids	128	0 (0%)	NA	Wild boar	Meat
Atanassova et al. (2008)	Suids	127	0 (0%)	NA	Wild boar	Carcass
Avagnina et al. (2012)	Suids	65	0 (0%)	NA	Wild boar	Carcass
Bachand et al. (2012)	Suids	11	0 (0%)	NA	Red river hog	Carcass
Bonardi et al. (2021)	Suids	64	5 (8%)	NA	Wild boar	Carcass
Cilia et al. (2021)	Suids	287	9 (3%)	NA	Wild boar	Edible offal
Citterio et al. (2014)	Suids	28	0 (0%)	NA	Wild boar	Carcass
Conedera et al. (2014)	Suids	341	19 (6%)	NA	Wild boar	Edible offal
Díaz-Sánchez et al. (2013)	Suids	333	4 (1%)	NA	Wild boar	Carcass
Eglezos et al. (2008)	Suids	217	3 (1%)	NA	Wild boar	Carcass
Elezi et al. (2013)	Suids	20	5 (25%)	NA	Wild boar	Meat
Floris et al. (2024)	Suids	34	0 (0%)	0 (0%)	Wild boar	Meat
Floris et al. (2024)	Suids	27	0 (0%)	0 (0%)	Wild boar	Edible offal
Fredriksson-Ahomaa et al. (2020)	Suids	130	0 (0%)	6 (5%)	Wild boar	Edible offal
Giorda et al. (2014)	Suids	171	16 (9%)	NA	Wild boar	Edible offal
Kabwang a Mpalang et al. (2013)	Suids	60	3 (5%)	6 (10%)	Warthog	Meat
Ludwiczak et al. (2019)	Suids	16	0 (0%)	NA	Wild boar	Meat
Mirceta et al. (2017)	Suids	210	4 (2%)	NA	Wild boar	Carcass
Orsoni et al. (2020)	Suids	37	0 (0%)	NA	Wild boar	Carcass
Paulsen and Winkelmayer (2004)	Suids	3	0 (0%)	NA	Wild boar	Carcass
Peruzy et al. (2019)	Suids	22	6 (27%)	7 (32%)	Wild boar	Meat
Peruzy, Cristiano, et al. (2022)	Suids	28	10 (36%)	10 (36%)	Wild boar	Meat
Peruzy, Murru, et al. (2022)	Suids	36	1 (3%)	1 (3%)	Wild boar	Carcass
Petrović et al. (2024)	Suids	425	4 (1%)	NA	Wild boar	Carcass
Ranucci et al. (2021)	Suids	120	3 (2%)	NA	Wild boar	Carcass
Razzuoli et al. (2021)	Suids	4335	260 (6%)	540 (12%)	Wild boar	Edible offal
Rîmbu et al. (2015)	Suids	28	3 (11%)	NA	Wild boar	Carcass
Siddi et al. (2023)	Suids	49	0 (0%)	NA	Wild boar	Carcass
Stella et al. (2018)	Suids	30	0 (0%)	NA	Wild boar	Carcass
Asakura et al. (2017)	Ruminants	120	0 (0%)	NA	Deer (not specified)	Meat
Atanassova et al. (2008)	Ruminants	95	0 (0%)	NA	Western roe deer	Carcass
Atanassova et al. (2008)	Ruminants	67	0 (0%)	NA	Red deer	Carcass
Avagnina et al. (2012)	Ruminants	69	0 (0%)	NA	Chamois	Carcass
Avagnina et al. (2012)	Ruminants	61	0 (0%)	NA	Western roe deer	Carcass
Avagnina et al. (2012)	Ruminants	56	0 (0%)	NA	Red deer	Carcass
Bachand et al. (2012)	Ruminants	45	0 (0%)	NA	Duikers	Carcass
Branciari et al. (2020)	Ruminants	64	0 (0%)	NA	Western roe deer	Carcass
Citterio et al. (2014)	Ruminants	69	0 (0%)	NA	Chamois	Carcass
Citterio et al. (2014)	Ruminants	86	0 (0%)	NA	Western roe deer	Carcass
Citterio et al. (2014)	Ruminants	89	0 (0%)	NA	Red deer	Carcass

**Reference**	**Animal group**	**Number of samples**	**Culture results**	**PCR results**	**Animal species**	**Matrix**
Citterio et al. (2014)	Ruminants	8	0 (0%)	NA	Mouflon	Carcass
Díaz-Sánchez et al. (2013)	Ruminants	295	1 (0%)	NA	Red deer	Carcass
Floris et al. (2024)	Ruminants	42	0 (0%)	0 (0%)	Chamois	Meat
Floris et al. (2024)	Ruminants	21	0 (0%)	0 (0%)	Western roe deer	Meat
Floris et al. (2024)	Ruminants	39	0 (0%)	0 (0%)	Red deer	Meat
Floris et al. (2024)	Ruminants	35	0 (0%)	0 (0%)	Chamois	Edible offal
Floris et al. (2024)	Ruminants	22	0 (0%)	0 (0%)	Western roe deer	Edible offal
Floris et al. (2024)	Ruminants	26	0 (0%)	0 (0%)	Red deer	Edible offal
Giorda et al. (2014)	Ruminants	48	1 (2%)	NA	Chamois	Edible offal
Giorda et al. (2014)	Ruminants	24	0 (0%)	NA	Western roe deer	Edible offal
Hingston et al. (2020)	Ruminants	25	0 (0%)	NA	Eurasian elk	Meat
Hingston et al. (2020)	Ruminants	2	0 (0%)	NA	Deer, species not specified	Meat
Kabwang a Mpalang et al. (2013)	Ruminants	63	2 (3%)	2 (3%)	African buffalo	Meat
Kabwang a Mpalang et al. (2013)	Ruminants	59	3 (5%)	3 (5%)	Duikers	Meat
Nkosi et al. (2022)	Ruminants	158	24 (15%)	NA	Impala	Meat
Paulsen and Winkelmayer (2004)	Ruminants	25	0 (0%)	NA	Red deer	Carcass
Paulsen and Winkelmayer (2004)	Ruminants	18	0 (0%)	NA	Western roe deer	Carcass
Paulsen and Winkelmayer (2004)	Ruminants	3	0 (0%)	NA	Chamois	Carcass
Paulsen and Winkelmayer (2004)	Ruminants	1	0 (0%)	NA	Mouflon	Carcass
Sauvala et al. (2019)	Ruminants	100	0 (0%)	4 (4%)	Eurasian elk	Carcass
Sauvala et al. (2019)	Ruminants	100	0 (0%)	0 (0%)	White-tailed deer	Carcass
Sauvala et al. (2023)	Ruminants	50	NA	0 (0%)	White-tailed deer	Meat

NA: not applicable

Table S8 | Overview of studies reporting the prevalence of STEC in game meat of wild hunted ungulates.

Reference	Animal group	Number of samples	Culture results	PCR results	Animal species	Matrix
Asakura et al. (2017)	Suids	128	0 (0%)	0 (0%)	Wild boar	Meat
Díaz-Sánchez et al. (2012)	Suids	73	3 (4%)	12 (16%)	Wild boar	Meat
Díaz-Sánchez et al. (2013)	Suids	310	12 (4%)	57 (18%)	Wild boar	Carcass
Floris et al. (2024)	Suids	24	0 (0%)	NA	Wild boar	Edible offal
Fredriksson-Ahomaa et al. (2020)	Suids	130	NA	43 (33%)	Wild boar	Edible offal
Kabwang a Mpalang et al. (2013)	Suids	60	0 (0%)	0 (0%)	Warthog	Meat
Magwedere et al. (2013)	Suids	2	NA	0 (0%)	Wild boar	Meat
Nüesch-Inderbinen et al. (2023)	Suids	25	3 (12%)	16 (64%)	Wild boar	Meat
Peruzy, Cristiano, et al. (2022)	Suids	28	4 (14%)	12 (43%)	Wild boar	Meat
Peruzy, Murru, et al. (2022)	Suids	36	4 (11%)	7 (19%)	Wild boar	Carcass
Asakura et al. (2017)	Ruminants	120	1 (1%)	1 (1%)	Deer (not specified)	Meat
Díaz-Sánchez et al. (2012)	Ruminants	69	5 (7%)	24 (35%)	Red deer	Meat
Díaz-Sánchez et al. (2013)	Ruminants	271	19 (7%)	68 (25%)	Red deer	Carcass
Floris et al. (2024)	Ruminants	34	0 (0%)	NA	Chamois	Edible offal
Floris et al. (2024)	Ruminants	18	0 (0%)	NA	Western roe deer	Edible offal
Floris et al. (2024)	Ruminants	24	0 (0%)	NA	Red deer	Edible offal
Haindongo et al. (2018)	Ruminants	41	NA	32 (78%)	Springbok	Carcass
Haindongo et al. (2018)	Ruminants	75	NA	52 (69%)	Gemsbok	Carcass
Haindongo et al. (2018)	Ruminants	5	NA	5 (100%)	Greater kudu	Carcass
Haindongo et al. (2018)	Ruminants	5	NA	5 (100%)	Wildebeest	Carcass
Kabwang a Mpalang et al. (2013)	Ruminants	63	0 (0%)	4 (6%)	African buffalo	Meat
Kabwang a Mpalang et al. (2013)	Ruminants	59	0 (0%)	0 (0%)	Duikers	Meat
Listorti et al. (2024)	Ruminants	289	28 (10%)	28 (10%)	Multiple species	Edible offal
Magwedere et al. (2013)	Ruminants	3	NA	1 (33%)	White-tailed deer	Meat
Magwedere et al. (2013)	Ruminants	20	NA	0 (0%)	Red deer	Meat
Magwedere et al. (2013)	Ruminants	2	NA	0 (0%)	Reindeer	Meat
Magwedere et al. (2013)	Ruminants	24	NA	0 (0%)	American bison	Meat
Nüesch-Inderbinen et al. (2023)	Ruminants	2	1 (50%)	1 (50%)	Chamois	Meat
Nüesch-Inderbinen et al. (2023)	Ruminants	27	4 (15%)	24 (89%)	Red deer	Meat
Nüesch-Inderbinen et al. (2023)	Ruminants	38	15 (39%)	36 (95%)	Western roe deer	Meat
Sauvala et al. (2019)	Ruminants	100	NA	16 (16%)	Eurasian elk	Carcass
Sauvala et al. (2019)	Ruminants	100	NA	12 (12%)	White-tailed deer	Carcass
Sauvala et al. (2023)	Ruminants	50	NA	9 (18%)	White-tailed deer	Meat
Thierry et al. (2020)	Ruminants	61	6 (10%)	NA	Rusa deer	Meat

NA: not applicable

Table S9 | Overview of studies reporting the prevalence of *Listeria* spp. in game meat of wild hunted ungulates.

Reference	Animal group	Number of samples	Culture results	PCR results	Animal species	Matrix
Atanassova et al. (2008)	Suids	127	7 (6%)	NA	Wild boar	Carcass
Citterio et al. (2014)	Suids	28	2 (7%)	NA	Wild boar	Carcass
Floris et al. (2024)	Suids	28	2 (7%)	3 (11%)	Wild boar	Edible offal
Fredriksson-Ahomaa et al. (2020)	Suids	130	40 (31%)	63 (48%)	Wild boar	Edible offal
Ludwiczak et al. (2019)	Suids	16	0 (0%)	NA	Wild boar	Meat
Paulsen and Winkelmayer (2004)	Suids	3	0 (0%)	NA	Wild boar	Carcass
Peruzy et al. (2019)	Suids	22	0 (0%)	0 (0%)	Wild boar	Meat
Rîmbu et al. (2015)	Suids	28	2 (7%)	NA	Wild boar	Carcass
Stella et al. (2018)	Suids	30	8 (27%)	NA	Wild boar	Carcass
Weindl et al. (2016)	Suids	49	2 (4%)	NA	Wild boar	Edible offal
Atanassova et al. (2008)	Ruminants	95	4 (4%)	NA	Western roe deer	Carcass
Atanassova et al. (2008)	Ruminants	67	3 (4%)	NA	Red deer	Carcass
Branciari et al. (2020)	Ruminants	64	0 (0%)	NA	Western roe deer	Carcass
Citterio et al. (2014)	Ruminants	69	3 (4%)	NA	Chamois	Carcass
Citterio et al. (2014)	Ruminants	86	2 (2%)	NA	Western roe deer	Carcass
Citterio et al. (2014)	Ruminants	89	3 (3%)	NA	Red deer	Carcass
Citterio et al. (2014)	Ruminants	8	0 (0%)	NA	Mouflon	Carcass
Floris et al. (2024)	Ruminants	34	0 (0%)	0 (0%)	Chamois	Edible offal
Floris et al. (2024)	Ruminants	22	0 (0%)	0 (0%)	Western roe deer	Edible offal
Floris et al. (2024)	Ruminants	26	0 (0%)	0 (0%)	Red deer	Edible offal
Hingston et al. (2020)	Ruminants	25	0 (0%)	NA	Eurasian elk	Meat
Hingston et al. (2020)	Ruminants	2	0 (0%)	NA	Deer, species not specified	Meat
Paulsen and Winkelmayer (2004)	Ruminants	25	0 (0%)	NA	Red deer	Carcass
Paulsen and Winkelmayer (2004)	Ruminants	18	0 (0%)	NA	Western roe deer	Carcass
Paulsen and Winkelmayer (2004)	Ruminants	3	0 (0%)	NA	Chamois	Carcass
Paulsen and Winkelmayer (2004)	Ruminants	1	0 (0%)	NA	Mouflon	Carcass
Sauvala et al. (2019)	Ruminants	100	7 (7%)	8 (8%)	Eurasian elk	Carcass
Sauvala et al. (2019)	Ruminants	100	6 (6%)	12 (12%)	White-tailed deer	Carcass
Sauvala et al. (2023)	Ruminants	50	NA	25 (50%)	White-tailed deer	Meat
Weindl et al. (2016)	Ruminants	45	0 (0%)	NA	Red deer	Edible offal
Avagnina et al. (2012)	Multiple	251	7 (3%)	NA	Multiple species	Carcass

NA: not applicable

Table S10 | Overview of studies reporting the prevalence of *Yersinia* spp. in game meat of wild hunted ungulates.

Reference	Animal group	Number of samples	Culture results	PCR results	Animal species	Matrix
Avagnina et al. (2012)	Suids	65	10 (15%)	NA	Wild boar	Carcass
Bancerz-Kisiel et al. (2016)	Suids	40	7 (18%)	NA	Wild boar	Carcass
Bonardi et al. (2021)	Suids	64	3 (5%)	NA	Wild boar	Carcass
Floris et al. (2024)	Suids	27	5 (19%)	NA	Wild boar	Edible offal
Fredriksson-Ahomaa et al. (2020)	Suids	130	2 (2%)	22 (17%)	Wild boar	Edible offal
Mignone et al. (2015)	Suids	2326	82 (4%)	NA	Wild boar	Edible offal
Modesto et al. (2021)	Suids	4890	126 (3%)	NA	Wild boar	Edible offal
Peruzy et al. (2019)	Suids	22	NA	3 (14%)	Wild boar	Meat
Peruzy, Cristiano, et al. (2022)	Suids	28	1 (4%)	21 (75%)	Wild boar	Meat
Peruzy, Murru, et al. (2022)	Suids	36	0 (0%)	14 (39%)	Wild boar	Carcass
Sannö et al. (2023)	Suids	32	NA	10 (31%)	Wild boar	Meat
Siddi et al. (2023)	Suids	49	3 (6%)	NA	Wild boar	Carcass
Avagnina et al. (2012)	Ruminants	69	1 (1%)	NA	Chamois	Carcass
Avagnina et al. (2012)	Ruminants	61	3 (5%)	NA	Western roe deer	Carcass
Avagnina et al. (2012)	Ruminants	56	4 (7%)	NA	Red deer	Carcass
Bancerz-Kisiel et al. (2016)	Ruminants	40	6 (15%)	NA	Western roe deer	Carcass
Bancerz-Kisiel et al. (2016)	Ruminants	32	4 (12%)	NA	Red deer	Carcass
Floris et al. (2024)	Ruminants	31	2 (6%)	NA	Chamois	Edible offal
Floris et al. (2024)	Ruminants	18	0 (0%)	NA	Western roe deer	Edible offal
Floris et al. (2024)	Ruminants	25	1 (4%)	NA	Red deer	Edible offal
Sauvala et al. (2019)	Ruminants	100	14 (14%)	6 (6%)	Eurasian elk	Carcass
Sauvala et al. (2019)	Ruminants	100	12 (12%)	9 (9%)	White-tailed deer	Carcass
Sauvala et al. (2023)	Ruminants	50	NA	0 (0%)	White-tailed deer	Meat

NA: not applicable

Table S11 | Overview of studies reporting the prevalence of Campylobacter spp. in game meat of wild hunted ungulates.

Reference	Animal group	Number of samples	Culture results	PCR results	Animal species	Matrix
Atanassova et al. (2008)	Suids	127	3 (2%)	NA	Wild boar	Carcass
Bachand et al. (2012)	Suids	11	0 (0%)	NA	Red river hog	Carcass
Fredriksson-Ahomaa et al. (2020)	Suids	130	0 (0%)	6 (5%)	Wild boar	Edible offal
Kabwang a Mpalang et al. (2013)	Suids	60	16 (27%)	16 (27%)	Warthog	Meat
Mignone et al. (2015)	Suids	2280	30 (1%)	NA	Wild boar	Edible offal
Peruzy, Cristiano, et al. (2022)	Suids	28	0 (0%)	0 (0%)	Wild boar	Meat
Peruzy, Murru, et al. (2022)	Suids	36	4 (11%)	4 (11%)	Wild boar	Carcass
Stella et al. (2018)	Suids	30	5 (17%)	NA	Wild boar	Carcass
Ziomek et al. (2023)	Suids	187	9 (5%)	NA	Wild boar	Edible offal
Ziomek et al. (2023)	Suids	55	10 (18%)	NA	Wild boar	Carcass
Atanassova et al. (2008)	Ruminants	95	0 (0%)	NA	Western roe deer	Carcass
Atanassova et al. (2008)	Ruminants	67	0 (0%)	NA	Red deer	Carcass
Bachand et al. (2012)	Ruminants	45	0 (0%)	NA	Duikers	Carcass
Kabwang a Mpalang et al. (2013)	Ruminants	63	6 (10%)	7 (11%)	African buffalo	Meat
Kabwang a Mpalang et al. (2013)	Ruminants	59	11 (19%)	13 (22%)	Duikers	Meat
Sauvala et al. (2019)	Ruminants	100	NA	6 (6%)	Eurasian elk	Carcass
Sauvala et al. (2019)	Ruminants	100	NA	2 (2%)	White-tailed deer	Carcass
Sauvala et al. (2023)	Ruminants	50	NA	1 (2%)	White-tailed deer	Meat

NA: not applicable
